# Learning attentional templates for value-based decision-making

**DOI:** 10.1016/j.cell.2024.01.041

**Published:** 2024-02-23

**Authors:** Caroline I. Jahn, Nikola T. Markov, Britney Morea, Nathaniel D. Daw, R. Becket Ebitz, Timothy J. Buschman

**Affiliations:** 1 Princeton Neuroscience Institute, Princeton University, Princeton, NJ 08540, USA; 2 Department of Psychology, Princeton University, Princeton, NJ 08540, USA; 3 Department of Neurosciences, Université de Montréal, Montréal, QC H3C 3J7, Canadas; 4 Lead contact

## Abstract

Attention filters sensory inputs to enhance task-relevant information. It is guided by an “attentional template” that represents the stimulus features that are currently relevant. To understand how the brain learns and uses templates, we trained monkeys to perform a visual search task that required them to repeatedly learn new attentional templates. Neural recordings found that templates were represented across the prefrontal and parietal cortex in a structured manner, such that perceptually neighboring templates had similar neural representations. When the task changed, a new attentional template was learned by incrementally shifting the template toward rewarded features. Finally, we found that attentional templates transformed stimulus features into a common value representation that allowed the same decision-making mechanisms to deploy attention, regardless of the identity of the template. Altogether, our results provide insight into the neural mechanisms by which the brain learns to control attention and how attention can be flexibly deployed across tasks.

## In brief

Jahn et al. show that the brain uses reward feedback to learn attentional templates that select task-relevant stimuli. Attended stimulus features were converted into a common value representation, allowing attentional control to generalize across tasks.

## Graphical Abstract

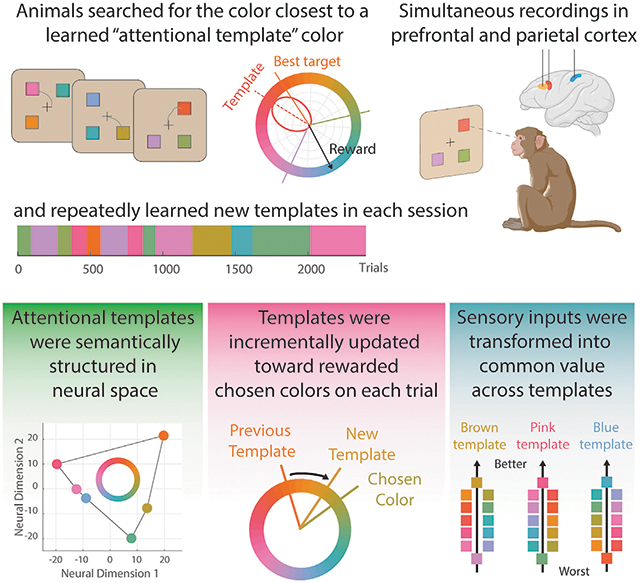

## INTRODUCTION

Cognitive control focuses on the stimuli, thoughts, and actions that are relevant to the current task. In particular, feature-based visual attention selects those stimuli that have task-relevant features. This is an everyday experience, e.g., we attend to yellow when hailing a taxicab in New York City. Feature-based attention is guided by an “attentional template” that contains the set of stimulus features that are relevant for the current task.^1–5^ Attentional templates are represented in the prefrontal and parietal cortex^6–8^: these regions are active when attention shifts toward specific features^9–11^ and lesioning (or inactivating) these regions impairs featural attention.^9,12–16^

As the task changes, so does the information that is relevant. The brain must flexibly adapt, continuously learning and relearning the optimal attentional template for each task. For example, one’s attentional template for finding taxis must be updated to beige in Berlin and red in Costa Rica. The neural mechanisms for learning attentional templates are largely unknown. One hypothesis is that templates are learned through reinforcement learning. Consistent with this, psychophysical studies have shown that reward can be used to learn new attentional templates.^17–23^ Furthermore, rewarded stimuli attract attention,^24,25^ and many value-learning studies are thought to also measure changes in attention toward rewarded stimuli.^26^ This makes sense; when learning a new task, rewarded stimuli or features are likely task-relevant and should be attended. However, most neurophysiological studies of attention provide the subjects with the template, and so we do not yet know how rewards shape attentional templates during learning.

Once learned, the attentional template influences sensory processing in order to select task-relevant stimuli.^8^ This is thought to occur in a top-down manner, with templates in the prefrontal and parietal cortex biasing sensory representations across the brain.^27–34^ By strengthening the neural representation of task-relevant features, attentional templates improve discrimination and influence decisions.^35,36^ Yet, it remains unclear how attention rapidly reconfigures sensory processing and decision-making as the attentional template flexibly changes between tasks.

To better understand how attentional templates are learned and flexibly bias decision-making, we trained monkeys to perform a visual search task that required repeatedly learning new attentional templates in a continuous stimulus space (color). Neural recordings in the prefrontal and parietal cortex found attentional templates were represented in both regions. Template representations were structured such that perceptually similar templates had similar neural representations. When the behavioral task changed, the attentional template was learned incrementally: on every trial, the neural representation of the template shifted toward rewarded features. Finally, we found that the attentional template transformed stimulus representations into a generalized value representation, allowing the animal to find the target stimulus, regardless of the stimulus’ color or the current attentional template. Altogether, our results provide insight into the neural mechanisms by which the control of attention can adaptively be learned and flexibly deployed across tasks.

## RESULTS

**Figure 1.**
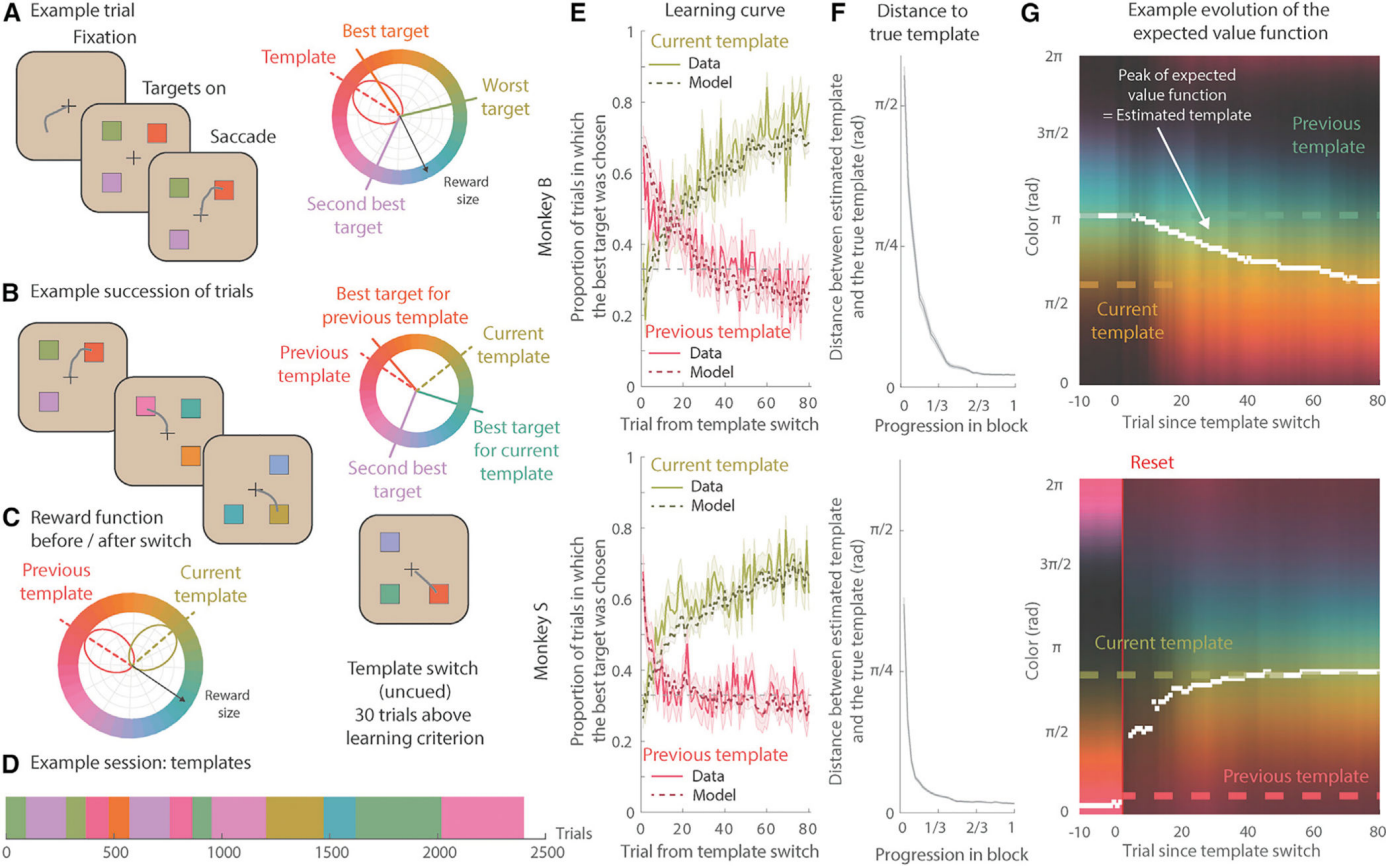
Attentional template learning task and behavior (A) (Left) Example trial. Randomly colored target stimuli were presented at three locations (randomly chosen out of four possible locations). Monkeys made a saccade to a target to “choose” it and receive the associated reward. (Right) Reward amount (number of drops; radial axis) varied as a function of the angular distance in color space between the chosen color and the template. (B) Example trial sequence. After monkeys reached a learning criterion (see STAR Methods), the template color changed. This switch was uncued. (C) Reward magnitude (radial axis) associated with a stimulus color (angular axis) changes when template changes. (D) Sequence of templates for an example session. Template color was pseudo-randomly chosen (see STAR Methods). (E) Learning curves for monkey B (top, 69 blocks) and monkey S (bottom, 102 blocks). The probability to choose the current best target increased after the template switch (green), whereas the probability of choosing the target that would have been best for the previous template decreased (pink). Shaded areas represent SEM. Solid and dashed lines represent the monkey’s and model’s behavior, respectively. Gray dashed line indicates chance. (F) Mean absolute angular distance between the animal’s estimated template (the color with the highest expected value according to the model) and the true template color decreased with learning (mean ± SEM across blocks in 30 bins of normalized block length). (G) Examples of expected value distribution during learning. Brighter colors indicate higher expected value, according to the model. Previous and current templates are indicated with dashed lines. White marker indicates the peak of the expected value function on each trial. After a switch in template, the estimated template either (top) smoothly drifted toward the current template or (bottom) reset to uniform before reemerging at the new template. Resets (red line) were triggered by a large RPE, which occurred after a large change in the template color (Figures S1D and S1E). See also Figures S1 and S2.

We trained two monkeys to perform a visual search task, where they repeatedly learned new attentional templates. On each trial, the monkeys searched a visual array of three randomly colored stimuli (Figures 1A and 1B; see STAR Methods for details). They selected one stimulus by making an eye movement to it and then received the associated reward. The amount of reward associated with each stimulus was inversely proportional to the angular distance between the color of the stimulus and the template color (Figure 1A, right; smaller distance equaled greater reward). Therefore, on each trial, the animals used their attentional template to search the array of stimuli in order to identify, and select, the stimulus with the “best” color while ignoring the two distracting stimuli. Overall, the monkeys performed the task well, monkey B/S selected the best available stimulus on 65%/67% of trials.

During each session, the template color changed repeatedly (Figures 1B–1D). The monkeys were never explicitly instructed as to the identity of the template color or when it changed. Instead, they learned through trial and error, using reward as feedback to update their internal model of the template. Both monkeys quickly learned new templates: the proportion of trials in which the animal chose the best stimulus increased over the first dozen trials (Figure 1E, solid green; performance was above chance after 15/7 trials for monkey B/S, p < 0.05, Bonferroni corrected one-sided binomial test). As the animals learned the new template, they forgot the previous one, reflected in a decreasing proportion of trials choosing the stimulus closest to the previous template (Figure 1E, solid pink).

On average, the template was constant for a block of ~100 trials (96 ± 53/88 ± 50 attempted trials, mean ± standard deviation, for monkey B/S, full range was 30–306 trials). The change in template was not cued and occurred when the animal reliably chose the best available color (performance ≥ 80%/85% for 30 consecutive trials for monkey B/S). This allowed the monkeys to learn between 5 and 16 different templates per session (see Figure 1D for an example succession of templates; 8/9 sessions and 8.6 ± 2.7/11.3 ± 3.4 templates per session for monkey B/S). Because the animals learned several templates, a stimulus feature that was the target on one trial could be a distractor on another. So, the animal had to use attention to search the array for the currently best stimulus on each trial.

To model the monkey’s ability to learn an attentional template, we extended the standard Q-learning reinforcement learning model to approximate a continuous circular value function^37^ (Figure S1A, see STAR Methods for details on validation). In this model, the attentional template was represented by the expected value of colors across the color wheel. On each trial, the model selected the stimulus with the largest expected reward. After receiving reward feedback, the model updated its estimated value function such that colors with a positive reward prediction error (+RPE; i.e., received more reward than expected) incrementally increased their value, whereas colors with a negative RPE (−RPE; i.e., received less reward) incrementally decreased their value (Figure S1B). In this way, the model captured the monkey’s ability to represent an attentional template, apply it to the stimulus array, and update the template based on feedback.

The model fit the monkey’s behavior well (Figure 1E, dashed; likelihood of chosen stimulus was 0.5826 ± 0.2635/0.5858 ± 0.2686 for monkey B/S). During learning, the probability that the animal and model chose the best option was highly correlated (p < 0.001, r(80) = 0.8908/0.8697 for monkey B/S). Similarly, the model captured the animal’s forgetting the previous template (p < 0.001, r(80) = 0.8687/0.8403 for monkey B/S). The model also suggested that monkeys used two different strategies to learn a new attentional template after a change (Figures S1C–S1F). Following a small change in the template color (<π/2), the animal incrementally updated their template (example shown in Figure 1G, top). However, following a large change in template color (>π/2), the model predicted that the animals “reset” their attentional template to be uniform across colors before relearning a new template (Figure 1G, bottom, see STAR Methods). This model fit behavior better than alternative models that did not rely on incremental learning (Figures S1G and S1H). The model also captured how choices were influenced by interactions between stimuli and the animal’s uncertainty about the template (Figure S2).

Overall, the animal’s behavior suggests that monkeys incrementally learned an attentional template and then used this template to guide their search and their decision to select a stimulus. With this foundation, we next aimed to understand how attentional templates are represented, updated, and used to guide decisions in the brain.

### Attentional templates were distributed across the parietal and prefrontal cortex

**Figure 2.**
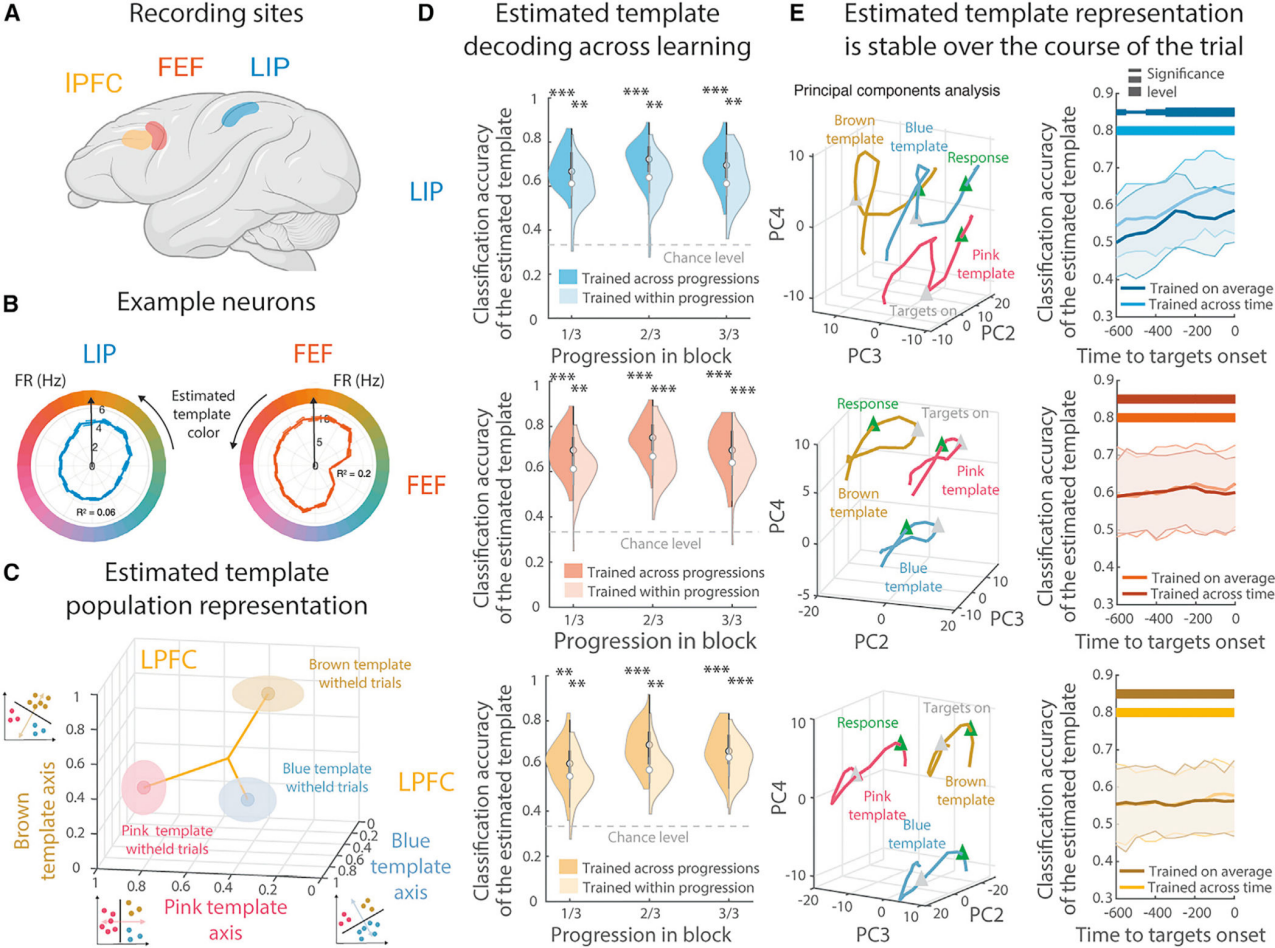
Distributed representation of the estimated attentional template in the parietal and frontal cortex (A) Recording sites. (B) Example LIP and FEF neurons encoding template. Radial axis is firing rate (mean ± SEM, dashed lines) across trials in a −600- to 300-ms window around the onset of the targets. Angular axis is color of estimated template (binsize=π/6, smoothing of three bins). (C) Neuron responses in LPFC, projected onto the vector normal to the hyperplane for the three classifiers trained to discriminate pink, brown, and blue templates (same time window as B). Insets show schematic of decoder. Ellipses represent the mean (central dot) and the SEM (shaded) of the projection across 100 bootstraps. Color indicates the estimated template on withheld trials. (D) Classification accuracy of the estimated template in (top) LIP, (middle) FEF, and (bottom) LPFC, for each third of the block, computed on withheld trials. Multiclass classifier was either trained (left half) across all or (right half) within each progression level in the block. Violin plot^38^: central white dot is the median, thick vertical gray bar represents the 25^th^ to 75^th^ quartile, and area represents the kernel density estimate of the data. **p ≤ 0.01, ***p ≤ 0.001. (E) Template representations are stable within a trial in (top) LIP (146 neurons), (middle) FEF (216 neurons), and (bottom) LPFC (475 neurons). Left: time course of response of neural population, projected into subspace of the second, third, and fourth principal component. Color indicates estimated template. Gray triangle indicates onset of targets. Green triangle indicates approximate time of response. (Right) classification accuracy of the estimated template in 300 ms windows either trained on the average activity in the full window (same as D and E) or trained for each window separately (100 bootstraps, mean and 95% confidence interval). Bar thickness above the data indicates significance level: thin, p ≤ 0.01 uncorrected; moderate, p ≤ 0.05 Bonferroni corrected across time (13 time points); and thick, p ≤ 0.01 Bonferroni corrected. See also Figure S3.

To understand how attentional templates are represented, we recorded from the prefrontal cortex, including lateral prefrontal cortex (LPFC, 492 neurons) and the frontal eye fields (FEF, 231 neurons), and parietal cortex (lateral intraparietal cortex, LIP, 167 neurons; Figure 2A). We leveraged the Q-learning behavioral model to estimate the animal’s latent representation of the template on each trial, taken as the color with the maximum expected value. Neurons in all three regions varied their activity as a function of the estimated template (Figures 2B and S3A; see Figure S3B for schematic of model variables). Across the population, a significant number of neurons smoothly encoded the color of the estimated template (Figure S3B; 30.54%/40.26%/30.08% in LIP/FEF/LPFC, all p ≤ 0.002, permutation test). Given the structure of the task, this is the simplest way to represent the attentional template—one merely selects the color that is closest to the template. It is also consistent with animals maintaining a single attentional template.^1–5^

In addition to the estimated template, a subset of neurons smoothly represented the expected value of individual colors (Figure S3B; 29.95%/16.02%/24.59% of neurons in LIP/FEF/LPFC, all p ≤ 0.002; see STAR Methods for details on model comparison). Such a continuous representation of expected value could allow one to estimate the expected value of any individual stimulus and, thus, calculate the RPE. Finally, a small proportion of neurons encoded the mean value of the trial or the chosen color (Figure S3B). Here, we focus our analyses on the representation of the estimated template, as it was the most strongly represented in all three regions (Figure S3B) and was the most compact representation of the template.

First, we were interested in understanding how the representation of the estimated template evolved during learning. Although all three regions represent the estimated template, one might expect differences during learning (e.g., early representation in LPFC and later representation in LIP). To track the evolution of template information, we trained a multi-class classifier to decode the estimated template from the neural population. Three linear classifiers were trained to discriminate one-third of the color wheel from the other two-thirds (Figure 2C, insets). All three classifiers decoded the estimated template on the majority of withheld trials in all three regions (Figure 2C for LPFC). The output of these classifiers was then fed into a shallow feedforward neural network to create a multi-class classifier (see STAR Methods). The multi-class classifier consistently decoded the template throughout learning in all three regions (Figure 2D, all p < 0.01, both across and within progression levels of learning, see STAR Methods). Reflecting its distributed nature, we did not find any differences in the timing or magnitude of the encoding of the estimated template across regions (Figures 2D and 2E).

The representation of the estimated template was stable during the trial. Plotting the neural activity in a low-dimensional space showed that temporal dynamics within the trial were largely independent from the estimated template representation in the LPFC and FEF (Figure 2E, left column, and S3D). Consistent with this, classifiers trained to decode the estimated template during one time point of the trial (e.g., during the fixation period) could decode estimated template representations later in the trial (e.g., during response) in the FEF and LPFC (Figure S3E). In the LIP, the coding of the estimated template was more dynamic, and the decoding accuracy increased specifically before and around the onset of the targets (Figure 2E, right column, and S3E). However, in all three regions, classifiers trained on neural activity averaged over the trial’s entire time period could decode the estimated template at any individual time period (Figure 2E, right column, all p < 0.01; differences between training across and within each time period were all p > 0.15). This suggests that dynamics in neural activity during the trial were orthogonal to the representation of the template (except for in the LIP, Figure 2E).

Together, our results suggest that attentional templates were stably represented across the frontal and parietal cortex, even as the animal learned (and relearned) new attentional templates. The stability of the representation may be important for allowing the template to guide attention on every trial. Next, we were interested in understanding the geometry of the template representation.

### Neural representation of attentional templates was structured

As with many sensory features, neurons in the sensory cortex that are selective to color tend to have smooth tuning curves, responding similarly to similar colors.^39^ At the level of the neural population, this smoothness creates a structured geometry such that perceptually similar colors are represented in similar ways in the brain.^40,41^ However, it is unknown whether control representations have similar structure.

Structured template representations could facilitate generalization, allowing for interpolation of new templates.^42^ An alternative hypothesis is that cognitive control representations are high dimensional^43^ and so a unique neural representation is learned for each attentional template (regardless of perceptual similarity). Unique representations may be easier to learn, as template representations are not constrained and high-dimensional representations would avoid interference between templates.^44^

**Figure 3.**
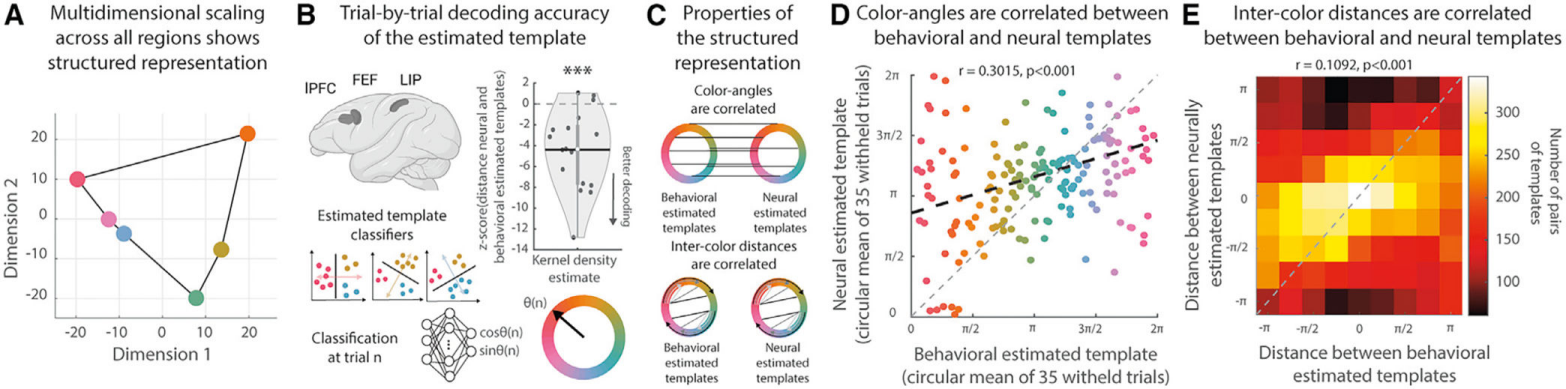
Neural attentional templates are structured (A) Representation of estimated templates in neural population. The population response to six template colors (independent bins, marker indicates color) into a two-dimensional space (defined by multidimensional scaling). LIP, 133 neurons; FEF, 216 neurons; LPFC, 459 neurons. (B) Schematic of decoding approach. Violin plot shows mean Z scored circular distance between the neural and behavioral estimated template on withheld trials for each session. ***p ≤ 0.001. (C) Schematic of properties of a structured representation. (D) Circular mean neural and behavioral estimated template across blocks, color is that of the mean behavioral estimated template (r = 0.3015, p < 0.001, 171 templates). Thick dashed line indicates the circular correlation; as the line lies below the diagonal, this suggests a bias in templates, possibly due to biases in color perception and/or memory.^45^ (E) Histogram of the circular distances between mean neural and behavioral estimated templates across blocks (r = 0.1092, p < 0.001, 14,535 pairs). See also Figure S4.

To begin to understand how template representations related to one another, we projected the neural representation of six different template colors in the prefrontal and parietal cortex into a reduced dimensional space (Figure 3A). Qualitatively, template representations reflected the semantic order of color space, suggesting that templates were represented in a structured manner in the prefrontal and parietal cortex.

To quantify the structure, we revised the template classifier used above to predict the angle of the estimated template along the color wheel (Figure 3B, see STAR Methods for details). Session-specific classifiers were trained on the activity of simultaneously recorded neurons from all three regions (increasing statistical power). We then applied the classifier to the first 35 trials of a new template color (all withheld from training) to test two predictions of a structured representation (Figure 3C).

First, if the neural representation of the template is structured, then it should match the behavioral template (which was structured by construct). Indeed, the neurally estimated template was correlated with the estimate of the behavioral model (r(5,838) = 0.1788, p < 0.001, circular correlation) and the distance between the neural and behavioral template was significantly less than expected by chance (Figure S4A, average absolute circular distance = 1.2662, p < 0.001, permutation test). This effect was consistent across sessions (Figure 3B; t(16) = −4.7694, p < 0.001, one-sided t test across sessions). Furthermore, the neutrally estimated color was correlated with the behaviorally estimated color across the spectrum of different templates (Figure 3D, r(171) = 0.3015, p < 0.001, circular correlation across blocks; Figure S4B, absolute circular distance = 1.1419, p < 0.001, permutation test). Finally, consistent with the neural template reflecting structure in the behavioral template, the decoding error of the neural model increased when the animal was uncertain about the attentional template (i.e., when the expected value function became more uniform, losing structure, Figures S4D and S4E).

Second, a structured neural representation should capture the semantic relationship between templates—templates close in perceptual space should also be close in neural space (Figure 3C, lower). Consistent with this, the distance between pairs of neutrally estimated templates predicted the distance between behaviorally estimated templates (Figure 3E; see Figure S4F for all pairs; r(14,535) = 0.1092, p < 0.001, circular correlation; Figure S4C, absolute circular distance = 1.4267, p < 0.001, permutation test).

Overall, our results suggest that frontoparietal cortex represents the monkeys’ internal model of the current attentional template in a structured fashion, with perceptually related templates represented in similar ways. Next, we were interested in understanding how the neural representation of the attentional template was learned.

### Attentional templates in the frontal and parietal cortex were learned through incremental updates

Previous work has found that the brain uses a variety of experience-driven (unsupervised),^46^ predictive (semi-supervised),^47^ and reward-driven (supervised),^48,49^ mechanisms to learn neural representations for perception and action. However, relatively little is known about how representations controlling attention are learned.

**Figure 4.**
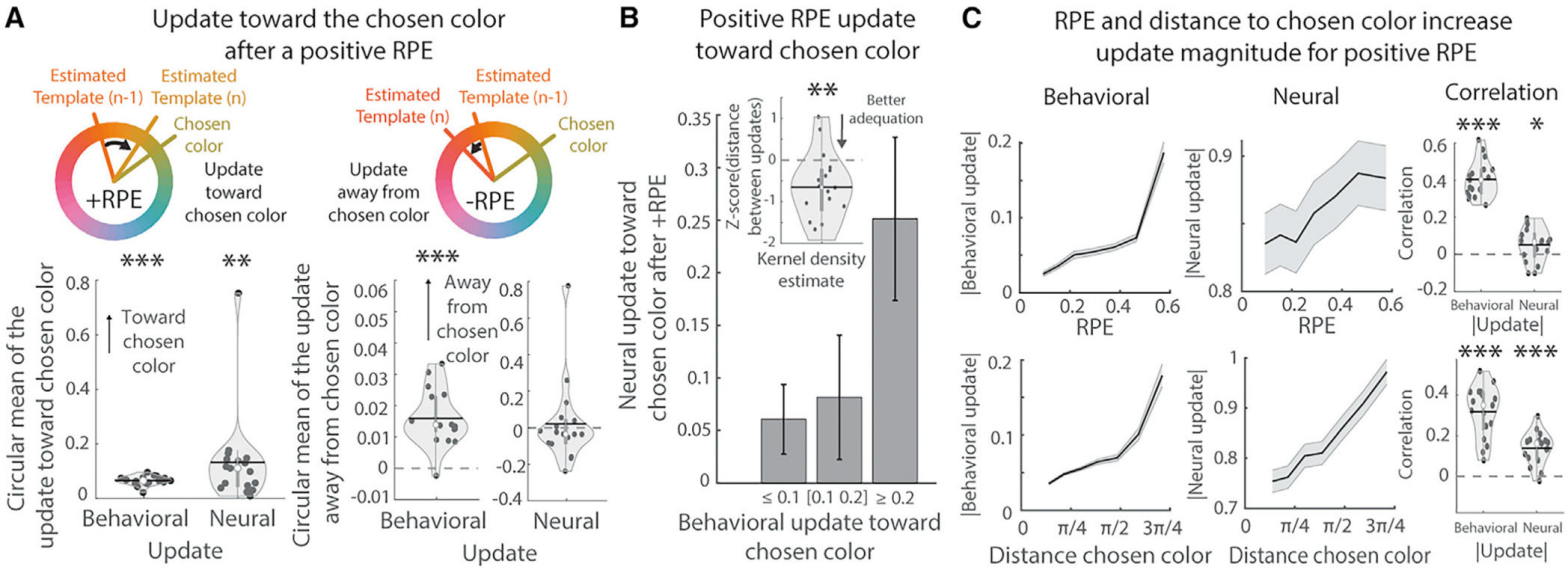
Neural attentional templates are incrementally updated in the structured space (A) (Left, top) Schematic of predicted change in template toward chosen color following a positive reward prediction error (+RPE). (Left, bottom) Circular mean of behavioral and neural updates toward the chosen color across sessions (all on withheld trials). (Right) Same as left, but for −RPEs, where the estimated template moves away from the chosen color. (B) Circular mean of neural update toward the chosen color (± SEM) after +RPE as a function of the size of the behavioral update (865, 268, and 170 trials in each bin). Inset shows violin plot of mean Z scored distance between the neural and the behavioral update toward the chosen color across validation trials for each session. (C) (Top row) mean (left) behavioral and (middle) neural update magnitude (± SEM) as a function of magnitude of +RPE (10 bins, smoothing of 4 bins) across all validation trials following +RPE. (Right) Violin plot of mean Pearson correlation between the update magnitude and the magnitude of +RPE across validation trials for each session. (Bottom row) same as top row, but for absolute distance between the estimated template before the update (i.e., at n-1) and the chosen color. For all panels, *p ≤ 0.05, **p ≤ 0.01, ***p ≤ 0.001. See also Figure S4.

The animal’s behavior suggests that templates may be learned using reinforcement learning in a continuous space (Figure 1E). If true, then this makes several predictions about how the attentional template should change in response to feedback. First, when the animal receives a reward that is higher than expected (+RPE), then the template representation should shift to be more similar to the chosen color. This effect was observed in the animals’ behavior across all trials (circular mean behavioral update was toward the chosen color; t(2,945) = 15.0389, p < 0.001, one-sided t test). This effect was also consistent across individual sessions (Figure 4A; t(16) = 15.8923, p < 0.001, one-sided t test). A similar effect was seen in the neural representation of the template (circular mean neural update was toward the chosen color; t(2,945) = 4.2168, p < 0.001, one-sided t test). Again, this effect was consistent across sessions (Figure 4A; t(16) = 3.2355, p = 0.0026, one-sided t test). Furthermore, the behavioral update predicted the neural update (p = 0.014, by permutation test on absolute circular distance), an effect that was consistent across sessions (Figure 4B, t(16) = −3.3410, p = 0.0021, one-sided t test).

A second prediction is that, if the neural representation of the template followed a reward-driven update rule, then the change in the representation should be larger when (1) the animal received a greater magnitude +RPE and (2) the distance between the previous template and the (rewarded) chosen color was greater. As predicted, the behavioral update depended on the +RPE magnitude (Figure 4C, top left; r(2,945) = 0.3676, p < 0.001, one-sided Pearson correlation), an effect that was consistent across sessions (Figure 4C, top right; t(16) = 17.0713, p < 0.001, one-sided t test on correlation coefficients). Although weaker, a similar effect was seen for neural updates: the overall effect was trending (Figure 4C, top-middle; r(2,945) = 0.0243, p = 0.0939, one-sided Pearson correlation) but the effect was consistent on individual sessions (Figure 4C, top right; t(16) = 2.2864, p = 0.0181, one-sided t test). Second, as predicted, the update magnitude was related to the distance between the template and chosen color for both behavioral and neural updates (Figure 4C, bottom; behavioral: r(2,945) = 0.2941, p < 0.001; neural: r(2,945) = 0.1422, p < 0.001, one-sided Pearson correlation). Again, these effects were consistent across sessions (Figure 4C, bottom right; behavioral: t(16) = 10.3213, p < 0.001; neural: t(16) = 7.1118, p < 0.001, one-sided t test).

Finally, in accordance with the template update being influenced by both +RPEs and the distance between template and chosen color, the greatest changes in template representations were observed after a reset, when learning was greatest (Figure S4G).

For negative RPEs (−RPE), the behavioral model showed a small shift in the template away from the chosen color (Figure 4A; t(2,669) = 14.3303, p < 0.001; across sessions: t(16) = 7.4167, p < 0.001, one-sided t test; effect was ~10x smaller than +RPE). Interestingly, we did not observe this in the neural data (t(2,669) = −2.1542, p = 0.9843; across sessions: t(16) = 0.3715, p = 0.3576, one-sided t test). And, unlike +RPEs, the magnitude of the update in the neural template was not related to −RPE magnitude nor distance between template and chosen color (Figure S4H). We do not think that the lack of update following a −RPE was because the learning rate was different: models that included different learning rates for +RPE and −RPE did not fit the behavior better than the simpler model with one learning rate (Δ Bayesian information criterion [BIC] = 467/406 for monkey B/S, see STAR Methods).

Altogether, these results suggest the representation of the estimated template in the frontoparietal cortex was learned through incremental updates following each trial. By repeatedly shifting the template toward stimuli with +RPEs, the template could evolve toward the optimal template color for that block of trials.

### The prefrontal and parietal cortex represented the value of individual stimuli in a way that generalized across templates

**Figure 5.**
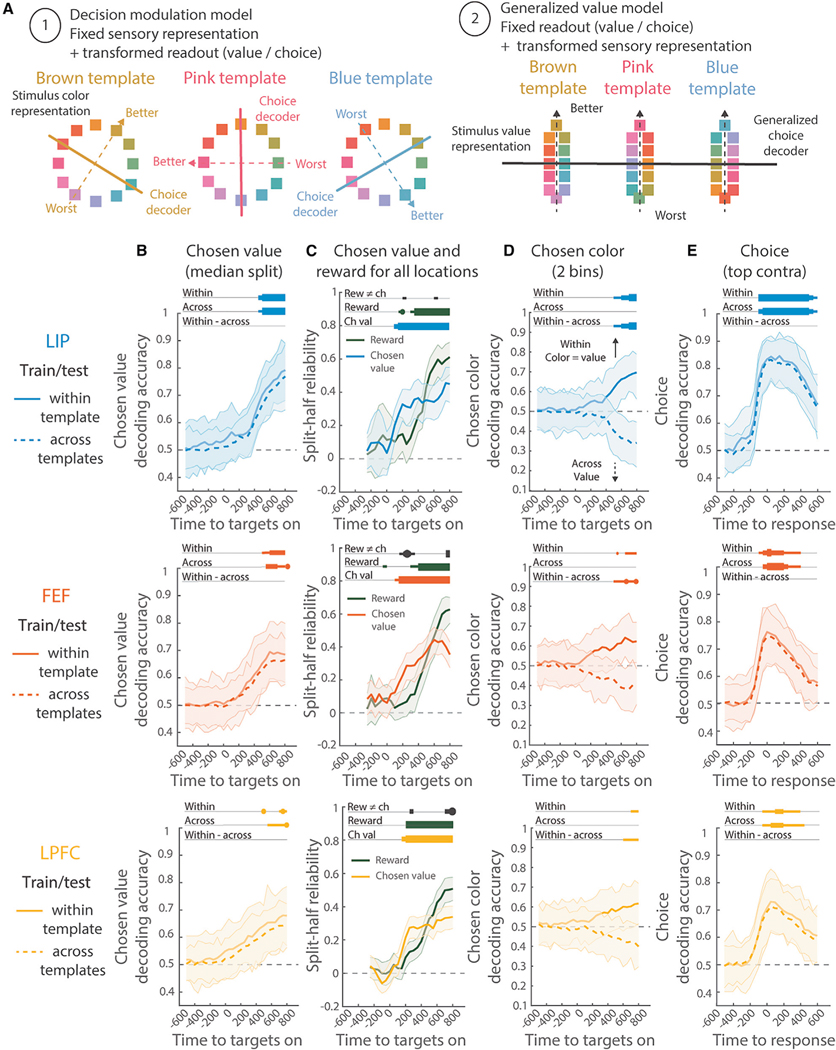
Re-mapping stimuli to support a generalized decision-making process across templates (A) Schematic of two hypotheses for how the attentional template supports decisions: (1) animals learn template-specific decision boundaries or (2) templates transform sensory information into a generalized value signal, allowing for a fixed decision readout. (B) Classification accuracy (with 95% confidence interval) of the chosen value over time (median split within each session) for (top) LIP, (middle) FEF, and (bottom) LPFC. Computed on withheld trials from the same template color bin as the training trials (within, solid line) or in a different template color bin (across, dashed line). Chance level was 1/2 (gray dashed line). (C) Time course of the mean split-half reliability (with 95% confidence interval) of the chosen value (colored by area) and reward (green) regressors computed across all locations. Bars indicate significance of chosen value and reward, and their difference (black): thin, p ≤ 0.05; moderate, p ≤ 0.01; and thick, p ≤ 0.05; Bonferroni corrected (22 time points). (D) Same as (B), but for classifiers trained to decode the chosen color (balanced for the estimated template, 2 bins). (E) Same as (B), but for decoding choice at the top contralateral location. For (B, D, and E), bar thickness indicates significance level: thin, p ≤ 0.01; moderate, p ≤ 0.05 Bonferroni corrected; and thick, p ≤ 0.01 Bonferroni corrected. Bonferroni correction was across time (27/27/23 time points for B, ,D and E) and locations (4 locations for E). See also Figure S5.

Our task requires combining the attentional template with sensory information to decide which stimulus to select. There are two hypotheses for how templates could guide decisions. First, in a “decision modulation” model, the decision boundary changes for each template, rotating to optimally discriminate stimuli close/far from the template boundary (i.e., those with high/low value; Figure 5A, left). The decision boundary could be learned incrementally, similar to the template, or chosen from a set of pre-determined decision boundaries.^50^ Alternatively, in a “generalized value” model, the attentional template acts on sensory representations to transform them into generic “value” signals (Figure 5A, right). This would require re-mapping stimulus responses for every template (e.g., both “pink” when the template is “red” and “teal” when it is “blue” are high value), but would have the advantage of only needing one, stable decision boundary.^51–53^ Several lines of evidence support the second hypothesis.

First, the value of the stimulus chosen by the animal was represented in a stable manner that generalized across templates. A classifier trained to discriminate between chosen stimuli with high and low expected values performed significantly above chance in all three regions (Figure S5A; this controls for movements). Critically, the same classifier performed above chance on trials with a different attentional template (Figure 5B). In fact, the classifier performed equally well on the template it was trained on and when tested on different templates (difference was not significantly different from zero, p ≥ 0.24, uncorrected, for all three regions, permutation test). Projecting attentional template and value representations into the same subspace reveals how this is the case: template information and value are represented independently from one another (Figure S5G). Similar results were found when controlling for potentially confounding variables, such as reward (Figure 5C; measured with split-half reliability,^54^ see STAR Methods for details). Chosen value representation occurred immediately after the presentation of the stimuli and preceded the reward representation (Figure 5C, p = 0.001 in FEF and p = 0.044 in LPFC, effect in LIP was weaker, p = 0.065, all Bonferroni corrected).

Second, in support of the generalized value model, the colors of the stimuli were not encoded in any of our recorded regions. Decoders trained on the neural population failed to decode the color of the stimulus at any location (Figure S5B) and failed to decode the color of the selected stimulus (Figure S5C, controlling for the estimated template color). This is consistent with very few or no individual neurons representing the color of the chosen stimulus (Figures S3B and S3C). In line with a re-mapping of the sensory representation for each attentional template, we found a weak representation of the chosen color within each template (Figure 5D; p = 0.03 Bonferroni corrected in the LIP, and trending in the FEF and LPFC, p = 0.02/p = 0.03 uncorrected, see STAR Methods for details). But these decoders did not generalize to different templates (Figure 5D, p ≥ 0.42 uncorrected for all three regions; decoding was worse than within the same template, p = 0.018 Bonferroni corrected in the LIP, p = 0.007 and p = 0.016 in FEF and LPFC, uncorrected). If anything, when comparing across templates, color decoding was below chance, as would be expected if the chosen color re-mapped when the template changed (as predicted by the generalized value model).

Finally, we tested whether a “decision” classifier, trained to discriminate whether a stimulus was chosen at each location, generalized across attentional templates. The animal’s decision was strongly encoded in all three regions (Figures S5D and S3F). Consistent with a static decision boundary, classifiers generalized across templates. In fact, classifiers performed equally well on withheld trials from the same template and other templates (Figure 5E; difference was not significantly different from zero, p ≥ 0.2232, uncorrected, for all three regions; see Figure S5E for all locations). It is important to note that these decision classifiers likely integrate both the decision process and the execution of the movement. Although movements may be expected to generalize across templates, the fact that we observed generalization at all time periods, including at the earliest moments before the saccade, suggests that the decision-making process also generalizes.

Altogether, these results are consistent with the hypothesis that the attentional template transforms sensory inputs into a generalized value representation in the prefrontal and parietal cortex. Generalized value representations could allow the same neural circuitry to decide which stimulus to select, regardless of the attentional template. This avoids the need to learn bespoke decision circuitry for each template and allows the brain to flexibly control attention in many different tasks.^51–53^

### Value representations transform from location-specific to global

Lastly, we wanted to understand how value representations support decisions and learning. Decisions involve comparing the value of stimuli across locations in order to identify, and select, the stimulus with the greatest expected value. This requires a “local” representation of the stimulus’ value at each location. However, learning the attentional template should be agnostic to the location of the stimulus and, thus, requires a “global” representation of value. To understand how value was represented, we fit a generalized linear model (GLM) to quantify the encoding of the value of chosen and unchosen stimuli “locally” (at each location) and “globally” (across locations; all comparisons used split-half reliability,^54^ see STAR Methods). Our analyses revealed that value representations were dynamic, initially local to each location before evolving into a global representation.

**Figure 6.**
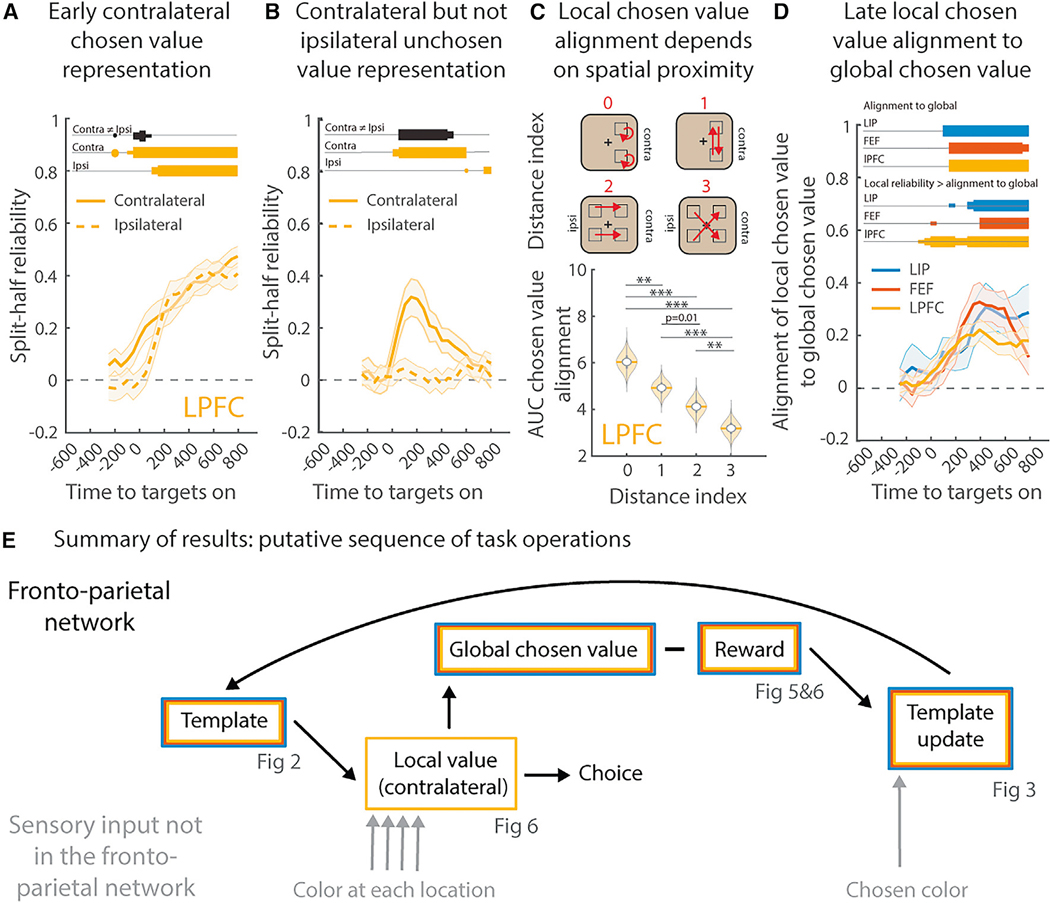
Value representations transformed from location-specific to global over time (A) Time course of the mean split-half reliability (with 95% confidence interval) of the chosen value at contralateral locations (contra) and ipsilateral locations (ipsi, dashed line) for LPFC. Bars indicate significance of each hemifield (yellow) and their difference (black). (B) Same as (A), but for unchosen value. (C) (Top) Schematic of distance index. (Bottom) Violin plot: mean area under the curve of the representation reliability (distance index = 0) or alignment across locations across the whole time period (22 time points, Figure S5E) for LPFC. Distribution across bootstraps. *p ≤ 0.05, **p ≤ 0.01, ***p ≤ 0.001 all Bonferroni corrected (6 pairs). See Figure S6G or LIP, and FEF and Figure S6L for the “early” time window alignment. (D) Same as (A), but for the correlation between the contralateral chosen value representation (shown in A and Figure S5A) and the global chosen value representation (shown in Figure 5C) for LIP, FEF, and LPFC. Upper bars (alignment to global) indicate the significance of the correlation disattenuation between the two vectors. Lower bars (local reliability > alignment to global) indicate whether the correlation between the two vectors is significantly lower than the local chosen value reliability. (E) Summary of results and putative sequence of task operations. Colored rectangles reflect involvement of a brain region in an operation (LPFC: yellow; FEF: red; LIP: blue). For all panels, bar thickness indicates significance level: thin, p ≤ 0.01; moderate, p ≤ 0.05 Bonferroni corrected across time (22 time points); and thick, p ≤ 0.01 Bonferroni corrected. See also Figure S6.

Immediately after stimulus presentation, value was represented in a location-specific manner. Value representations were initially restricted to the contralateral hemifield in the LPFC (Figures 6A and 6B; a similar, but weaker, effect in the FEF and LIP, Figures S6A and S6B). In fact, there was almost no encoding of the value of unchosen ipsilateral stimuli in any region (Figures 6B and S6B–S6D; similar results were found when considering the receptive field of neurons, Figures S6E–S6G). Further reflecting a localized representation, the neural representations of the value of chosen stimuli were more similar for locations that were closer together in physical space (Figures 6C and S6H–S6L, measured as the correlation in beta weights, permutation test).

If these initial, location-specific representations of value provide the input to the decision-making process, then they should precede the decision itself. Consistent with this, the values of chosen and unchosen stimuli were represented in a similar manner in the LPFC, reflecting the fact that location-specific value preceded the decision (measured as a correlation of beta weights, p = 0.0341, permutation test, Bonferroni corrected; 200 ms window centered on 150 ms after the onset of the targets, see STAR Methods). Similar, but weaker, results were seen in the LIP and FEF (p = 0.094/0.0598, uncorrected).

After a delay, the LPFC represented the value of the chosen stimulus, regardless of location (Figure 6A). This suggests that the value of the chosen stimulus was initially local but evolved into a global representation that generalized across locations. Consistent with this, the local representation of value slightly preceded the global representation (local: first significant ~50 ms in the LPFC, Figure 6A; global: 250 ms, Figure 5C; all Bonferroni corrected). Furthermore, the initial local representation of the value of the contralateral stimuli transformed over time, until it was aligned with the global representation (Figure 6D, measured as correlation in GLM beta weights; significant when the global representation emerges, 100/150/150 ms after the onset of the targets in LIP/FEF/LPFC, permutation test). This alignment was seen across locations, such that the global representation encoded the value of the chosen stimulus at all locations (Figures S6H–S6L).

Altogether, Figures 5 and 6 show that the attentional template transformed each stimulus into a local representation of the value at that location. This local representation is necessary for decision-making processes to determine where to move one’s eyes. Over time, the local representation transformed into a global, location-agnostic representation (particularly in the LPFC, Figure 6), possibly to support learning.

## DISCUSSION

Attention selects task-relevant sensory information for processing and for action.^8^ It is guided by an attentional template that represents the set of stimulus features that are currently relevant.^2–4^ Here, we sought to understand how the brain learns attentional templates and then uses them to influence sensory processing and drive decision-making. To this end, we trained two monkeys to perform a visual search task that required them to repeatedly learn new attentional templates (Figure 1). In brief, we found that the current attentional template was represented in a distributed fashion across the prefrontal and parietal cortex (Figure 2). Template representations were structured, with related templates (nearby in the color wheel) represented in a similar manner (Figure 3). During visual search, the template acted on sensory representations such that the feature of the stimulus was converted into a value representation (Figure 5). Value representations generalized across templates, allowing the same decision process to be used across all templates (Figure 5). To facilitate the decision of where to look, the value of each stimulus was represented in a topographic manner, with independent local value representations at each stimulus location (Figure 6). Once a stimulus was chosen, its local value transformed into a global representation shared across all locations (Figure 6), which could allow the brain to calculate a global reward prediction error. Finally, we found that the global reward prediction error was used to update the attentional template, such that attention shifted toward rewarded features (Figure 4). Altogether, our results provide a broad understanding of how attentional templates are learned and how they are used to guide eye movements in the frontal and parietal cortex (Figure 6E).

### Attentional templates are represented in a structured manner across the frontal and parietal cortex

Attentional templates were represented in a distributed network, suggesting that attention emerges from the dynamic interaction between the prefrontal and parietal cortex.^28–35^ We found some evidence that the LPFC may play a leading role in featural attention: the representation of the value of unchosen stimuli was stronger in the LPFC than FEF (Figure S6C, p = 0.0041 Bonferroni corrected, permutation test, see STAR Methods for details) and peaked earlier in the LPFC and LIP than the FEF (Figure S6D, LIP: p = 0.0217, LPFC: p = 0.0355, permutation test, see STAR Methods for details). Future work is needed to causally test this hypothesis—inactivating the LPFC impairs a monkey’s ability to choose a target based on feature-based attention,^9,55^ but it is unknown whether similar effects occur when inactivating parietal regions.

Our results suggest that the neural representation of attentional templates were structured, with perceptually similar colors represented by similar patterns of neural activity (Figure 3). Structured template representations may have several advantages. First, by matching the structure of sensory representations, moving through the space of control representations would have a smoothly varying effect on sensory representations. For example, a neuron encoding a red template, which biases red representations in the sensory cortex, would have a graded response to pink and purple. Second, structured representations could facilitate learning. When template representations vary smoothly, then updating the value of a single color should have a graded effect on nearby colors. Third, it could allow for generalization to new templates through interpolation. For example, a new “rose” template could be inferred to lie between red and pink. One cost of a structured template is that, for a given feature, the brain may be limited to a single attentional template, a constraint suggested by psychophysical studies^56^ (but see Hollingworth and Beck^57^).

Future work is needed to understand how structure emerges. Structure could have been acquired during training, either because rewards were graded for nearby colors or as the animals learned the general “meta” structure of the task.^58–60^ Alternatively, structured organization could have been inherited from the sensory cortex. Even when passed through random connections, neural representations maintain their local topography.^44^ Consistent with structure being innate, spatial representations are structured, even when animals are first learning a working memory task.^61^

### Attentional templates can be incrementally learned through reward-driven reinforcement learning

Psychophysical studies have shown the importance of rewards in shaping attentional templates.^17–23^ Reward modulates the representation of stimuli throughout the brain, including in the prefrontal and parietal cortex,^22^ and can lead to sharpening of tuning responses in the visual cortex (similar to attention).^62^ Our work builds on this foundation to study the neural mechanisms by which reward can shape attentional templates. Both behavioral modeling and neural results suggest that the brain uses reinforcement learning to update the internal model of the attentional template (Figure 4). These results are consistent with the large body of work showing reward learning can modulate the value of individual (discrete) representations,^63^ including updating the belief about the current task.^35,64,65^ Our work extends this to the continuous domain and provides insight into how new control representations could be learned.

Future work is needed to detail the mechanisms of learning. Learning the value of individual stimuli is thought to rely on the representation of reward prediction error in dopaminergic neurons.^66,67^ Given the strong dopaminergic innervation of the prefrontal cortex, a similar mechanism may act to update control of internal and cognitive states.^68^

### Creating a “priority map” for a generalized decision-making process

Despite being the task-relevant sensory feature, the color of stimuli was not represented in the prefrontal or parietal cortex during our task (Figures S3B, S3C, 5D, S5B, and S5C). This is not because these regions are not sensitive to color: stimulus color information has previously been found in all three regions.^41,69–72^ Instead, featural attention re-mapped sensory information into a value representation that generalized across templates. In this way, the prefrontal and parietal cortex represented a priority map of the visual scene.

Priority maps capture the priority of each stimulus in the visual field.^4,73–75^ This map can then be used to guide attention and decisions—in our task, the monkey simply selects the stimulus with the highest priority. These maps often reflect bottom-up, external characteristics of stimuli (e.g., size, brightness, etc.). Indeed, previous work has found priority maps of visually salient stimuli are represented in both the parietal and prefrontal cortex.^73,75^ However, as is the case in our task, priority maps can also integrate top-down, goal-directed attention to specific stimulus features.^4^ Our results show how attention integrates the current attentional template with sensory drive to form a map of the value of stimuli in the contralateral hemifield of the visual array. This map is then used to guide visual search and make a decision as to where to saccade in the visual scene (a form of overt attention). Importantly, because attention mapped sensory stimuli into a common value representation, the same priority map could be used to make decisions for all attentional templates.

Future work is needed to understand the mechanisms that generate a map of stimulus value. One hypothesis is that attention selectively increases the response of neurons representing stimuli matching the attentional template.^73,75^ By summing the activity of the neural population representing each location in space (across features), one could estimate how closely the stimulus at each location matched the attentional template (i.e., that location’s value). This would result in a priority map. Winner-take-all dynamics between locations on the priority map could select the location with the greatest priority, driving attention (overt or covert) to that location.^73^

### A common framework for attention, reward learning, and value-based decision-making

Attention, reward-learning, and value-based decision-making are closely interwoven cognitive operations. Our task requires top-down, selective attention as the animals must use the template to perform a covert visual search for a matching stimulus while filtering out distractors (both hallmarks of attention). However, the task also requires value-based decision-making, as the animals must use reward outcomes to update their internal representation of the attentional template. Although these concepts have largely been studied independently, our results show that integrating these concepts can deepen our understanding of both attention and decision-making. In our task, the mechanisms supporting learning attentional templates parallel the reinforcement learning mechanisms observed in simpler associative learning. Furthermore, our results show how value-based representations can allow the brain to generalize decision-making across a variety of attentional templates. These results suggest that attention and value-based decision-making may be overlapping, with a shared set of neural mechanisms.

### Beyond attention, implication for learning new tasks

Attention is one form of cognitive control: the control of sensory processing. Our results may extend to other forms of cognitive control. For example, we found attentional templates are structured such that semantically related tasks are represented in similar ways in the neural population. However, perception is not alone in being structured; previous work has shown that memories, spatial layouts, cognitive maps, and conceptual knowledge are also represented in a structured manner.^41,76–80^ Given this, cognitive control may be structured in other domains. This suggests that there may be one or more continuous “task spaces” in which smoothly changing the activity in the neural population may allow the brain to navigate through semantically related cognitive control states. As noted above, this could allow one to generalize to new situations by interpolating within the task space.

In addition, our results suggest that cognitive control can be learned through reinforcement learning, providing neural evidence for incremental learning of tasks.^60^ Such a mechanism could learn cognitive control representations *de novo*, without any relationship to other tasks. However, if task representations exist within a continuous task space, then incremental learning could move cognitive control states through this space in order to flexibly adapt to a changing environment. This could be combined with other mechanisms for learning cognitive control. For example, when in a new situation, one might use contextual cues to recall a generally appropriate task representation from previous, similar experiences (e.g., searching for a particular color car when looking for a cab). Incremental learning could then optimize the task representation for the current situation.

### Limitations of the study

Although our study provides insight into the learning and use of attentional templates, it has several limitations. First, although our behavioral model captured much of the monkey’s behavior, it was not perfect. For instance, the model could not account for the update in the neural template following −RPEs. Future work is needed to test whether this is because −RPEs had a small effect that may be hard to detect or whether a different mechanism responds to −RPEs (e.g., exploration).^81^ Furthermore, although the behavioral model suggests that the previously chosen color influenced choices, we did not find correlates of this representation in prefrontal or parietal neurons. In addition, future work is needed to understand how choices emerge from the interaction of several processes (e.g., top-down attention, episodic memory, biases, etc.) and how these processes are computed by the brain. Finally, our trial-by-trial estimate of the neural estimated template was noisier than our behavioral estimate (Figure 3). This may reflect a limited number of neurons and/or trials recorded on each day. Recent methodological advancements in simultaneously recording large numbers of neurons are likely to improve our ability to uncover trial-by-trial dynamics.

## STAR★METHODS

### RESOURCE AVAILABILITY

#### Lead contact

Further information and requests for resources and reagents should be directed to and will be fulfilled by the lead contact, Timothy J. Buschman (tbuschma@princeton.edu).

#### Materials availability

This study did not generate new unique reagents.

#### Data and code availability

- Both the code and neural data supporting each figure are publicly available online at https://doi.org/10.5281/zenodo.10529801.
- Original analysis code and behavioral and neural data are all publicly available via https://doi.org/10.5281/zenodo.10529801.
- Any additional information required to reanalyze the data reported in this paper is available from the lead contact upon request.

### EXPERIMENTAL MODEL AND STUDY PARTICIPANT DETAILS

#### Non-human primates

All experiments were conducted with two adult male rhesus macaques (monkey B, 13 kg, and monkey S, 9 kg).

### METHOD DETAILS

#### Ethics

All experimental procedures were approved by the Princeton University Institutional Animal Care and Use Committee and were in accordance with the policies and procedures of the National Institutes of Health.

#### Task and behavior

Stimuli were presented on a Dell U2413 LCD monitor positioned at a viewing distance of 58 cm. The monitor was calibrated using an X-Rite i1Display Pro colorimeter to ensure accurate color rendering. The task consisted in choosing one of three colored targets on a screen. These three colors were randomly selected from 100 evenly spaced points along an isoluminant circle in CIELAB color wheel.

The amount of reward R (in number of liquid drops) that a target was worth depended on the distance between its color θ (in radian) and the template in the color wheel. The value function was a von-mises (normalized circular gaussian) of fixed concentration κ that was set to 2.5:

(Equation 1) $$R(\theta )=R_{max}.\frac{e^{\kappa .\text{cos}(\theta -template)}}{2\pi I_{o}(\kappa )}$$

Where $I_{o}(\kappa )$ is the modified Bessel function of the first kind of order 0, such that the distribution sums to unity:

(Equation 2) $$2\pi .I_{o}(\kappa )=\int_{-\pi }^{\pi }e^{\kappa .\text{cos}(\theta )}d\theta$$

R was the reward size (number of drops) and was rounded to the nearest unit. The maximum size of the reward $R_{max}$ was fixed for monkey S (6 drops) and increased on each block for monkey B (12 drops + 1 drop for each completed block). The template color was fixed during a block such that subjects could learn it by trial and error, by choosing a color, receiving a feedback (reward size) and updating the expected value for each color. The template color was randomly selected at the start of each block. To increase the likelihood of sampling from all colors during a session, we temporarily penalized the colors near already selected colors during the selection process such that they were less likely to be selected again.

At the start of each trial, the monkeys fixated a central cross on the screen (distance to screen was approximately 65cm, screen diagonal was 68.58cm). After a delay of 350±50ms, three colored disks (of radius 1.5 visual degrees) appeared in three out of four possible locations (45, 135, 225, and 315 degrees from vertical with a 5 visual degrees eccentricity). The colors were separated by at least π/6 rad on the color wheel. Monkeys had 2000ms to select a target by making a saccade to it and maintaining fixation for at least 50ms. After 100ms, monkeys received the reward size (number of drops) associated with the chosen target. Only the chosen target stayed on screen while the reward was delivered. After 350/500ms for monkey B/S, a new trial started. If monkeys broke fixation before the onset of the targets or if they did not make a direct saccade to a stimulus, the screen changed color and a time-out of 200/500ms for monkey B/S was added to the inter-trial interval. In 20/5% of trials for monkey B/S, the size of one of the targets was bigger (radius was increase to 2.25 visual degrees) or smaller (radius was decreased to 0.75 visual degrees) for the duration of the display. This was random and did not predict reward or target location.

The template color was constant for a block of trials, allowing the monkey to learn and use the attentional template. The block (and the template color) would change when the monkey chose the target with the highest reward (i.e., the ‘best’ target) on 80/85% of 30 consecutive trials for monkey B/S. These template switches were uncued; the monkey could only detect the change through reward feedback. Blocks lasted between 80 and 575 trials (monkey B: 69 blocks, 138.2 ± 56.8 trials per block, monkey S: 102 blocks, 199.4 ± 114.1 trials per block). This was longer than the time it took monkeys to learn the template because it includes trials when the monkey broke fixation or did not make a direct saccade. To ensure a certain number of trials for data analysis, all blocks had at least 35 attempted trials before switching.

#### Behavioral model

We modelled the animal’s choice using a Q-learning model with function approximation.^37^ As in standard reinforcement learning models, the subject’s goal is to estimate the expected value v of each color θ in order to choose the best available color on each trial. Here, the state space is very large (100 colors) but continuous and structured according to the color wheel, so subjects can generalize what they learn about a color to the nearby colors (those that are closest in the color wheel). Hence, the expected value function can be represented not as a table (a value for each color) where each color is independent from the others, but as a parametrized function of N weight vectors with N being much less than the number of colors. In our case, the state space is represented by N equally distant radial basis functions $x_{i}$ that are von-mises functions of concentration κ and centered on $\mu_{i}$ (with $\mu_{1}=0$ rad):

(Equation 3) $$x_{i}(\theta )=\frac{e^{\kappa .\text{cos}\left(\theta -\mu_{i}\right)}}{2\pi I_{o}(\kappa )}$$

where $I_{o}$ is described in Equation 2. The parameter κ controls the degree of generalization during learning, the smaller κ, the more generalization. The expected value function v at trial n is a linear function of the weights $w_{i}$ and the radial basis functions $x_{i}$:

(Equation 4) $$v(\theta ,n)=\sum_{i=1}^{N}w_{i}(n).x_{i}(\theta )$$

The weights were initialized at 0 at the start of the session. After receiving an amount of reward R for choosing the color $\theta_{chosen}$, all the weights $w_{i}$ are updated according the learning rate α, the reward prediction error (RPE) and the distance between their centers $\mu_{i}$ and $\theta_{chosen}$:

(Equation 5) $$w_{i}(n+1)=w_{i}(n)+\alpha .RPE(t).x_{i}\left(\theta_{chosen}\right)$$

Where:

(Equation 6) $$RPE(n)=R\left(\theta_{chosen},n\right)-v\left(\theta_{chosen},n\right)$$

In this frame, the expected value function can be estimated for all colors at each trial.

We tested two variants of this model. In the No Reset model, the update follows Equation 5, and the uncued template switches are not detected. In the Reset model, the weights $w_{i}$ can be reset to uniform (all zeros) before doing the update. This occurred after a surprising event, when the absolute value of the RPE is above a threshold. The reset fraction $k_{reset}$ was:

(Equation 7) $$k_{reset}(n)=\frac{1}{1+e^{\beta_{reset}.(|RPE(n)|-Reset\;threshold(n))}}$$

where $\beta_{reset}$ was set to 10^4^ such that $k_{reset}$ was 0 or 1. Consecutive resets were penalized by increasing the *Reset threshold* based on the number of trials since the last reset (*# trials since last reset*). The smaller the *volatility* parameter, the greater the penalization:

(Equation 8) $$Reset\;threshold(n)=\frac{{Reset\;threshold}_{0}}{\text{tanh}(volatility.\#\;trials\;since\;last\;reset(n))}$$

For this second model the full update rule was:

(Equation 9) $$w_{i}(n+1)=\left(1-k_{reset}\right).\left(w_{i}(n)+\alpha .RPE(n).x_{i}\left(\theta_{chosen}\right)\right)+k_{reset}.R\left(\theta_{chosen},n\right).x_{i}\left(\theta_{chosen}\right)$$

At each trial n, we computed the expected value EV of each option θ by adding potential biases for the location of the option (Location(I)=1 at the location of the option and 0 otherwise), whether the size of the option was bigger or smaller than the standard size $\left(Size(s)=1\right.$ if the option was smaller (s=1) or bigger (s=2) and 0 otherwise), the proximity to a preferred colored $\theta_{pref}$ and the proximity to the previously chosen color $\theta_{prev\;chosen}$:

(Equation 10) $$EV(\theta ,n)=v(\theta ,n)+\sum_{l=1}^{4}location\;{bias}_{l}.Location(l)+\sum_{s=1}^{2}size\;{bias}_{s}.Size(s)+pref\;color\;bias.\left(\pi -\left|d\left(\theta ,\theta_{pref}\right)\right|\right)\;\;\;\;+prev\;color\;bias.\left(\pi -\left|d\left(\theta ,\theta_{prev\;chosen}\right)\right|\right)$$

Where d is the angular distance:

(Equation 11) $$d\left(\theta ,\theta_{0}\right)=mod\left(\theta -\theta_{0}+\pi ,2\pi \right)-\pi$$

We added these biases to capture non-value learning processes such as: choosing a specific location regardless of the stimulus presents (location biases), being influenced by irrelevant but salient features (size of the stimulus) and repeating the same choice (choosing the option closest to the previous selected color) regardless of the reward. The choice probability P for each target $\theta_{j}$ was computed using the softmax rule:

(Equation 12) $$P\left(\theta_{j}\right)=\frac{e^{EV\left(\theta_{i}\right)/\beta }}{\sum_{i=1}^{3}e^{EV\left(\theta_{i}\right)/\beta }}$$

β was set to 0.3 for both monkeys.

#### Behavioral model fit and selection

The No Reset model had 10 parameters and the Reset model had 12 parameters. Parameters were first fit using the Bayesian Adaptive Search (BADS) toolbox to get a first estimate of the parameters (https://github.com/lacerbi/bads). These parameters were used for the initialization of the fit with the Variational Bayes Monte-Carlo (VBMC) toolbox (https://github.com/lacerbi/vbmc).^82^ VBMC is an approximate inference method to fit and evaluate computational models. Specifically, it computes the approximate lower bound of the log model evidence (or log Bayes factor) for model comparison. We fit the models by first initializing the parameters using the BADS estimates (see Table S1). If the solution did not converge then we performed additional runs that were initialized using the posterior estimated on the previous run. All solutions converged.

Parameters were estimated by sampling the posterior distribution 3.10^5^ times.

We fit a series of models with N varying from 3 to 8 radial basis functions and compared them using the Bayesian Information Criterion (BIC) and the lower bound of the log model evidence. Both statistics reached an asymptote for N=6 for both monkeys (see Figure S1F) so this model was selected. Allowing for resets in the template improved the model’s accuracy (monkey B: ΔBIC=73, N=9,536 trials, monkey S: ΔBIC=895, N=20,338 trials; with 6 radial basis functions; Figure S1F) and so resets were included in the model. Unfortunately, the number of resets was too few to reliably identify reset related neural responses (113 reset events corresponding to 0.4% of trials across both monkeys). Table S2 contains the estimated parameters for each monkey.

Critically, in this formulation of the model, the learning rate can trade off with the concentration of the radial basis functions (κ) as κ relates to the height of the radial basis function (which has an area under the curve of 1) and a higher κ will correspond to a lower learning rate. This was observed in the posterior likelihood of the parameters which are negatively correlated. This means that we cannot identify the value of each parameter independently.

On each trial, the ‘estimated template’ was computed as the color with highest value:

(Equation 13) $$estimated\;template(t)={argmax}_{\theta \in [-\pi \pi [}(v(\theta ,t))$$

The entropy of the template was computed by transforming the value function in a strictly positive probability distribution:

(Equation 14) $$PV=\frac{V-\text{min}(V)+\frac{1}{N}}{\int_{\theta =-\pi }^{\pi }\left(V(\theta )-\text{min}(V)+\frac{1}{N}\right)d\theta }$$

Where V is the value function and N is the number of possible colors (values of θ, 100).


(Equation 15) $$entropy=-\int_{\theta =-\pi }^{\pi }PV(\theta ).\text{log}(PV(\theta ))d\theta$$

The biases (including for the previous chosen color) had only a marginal effect on choices. They drove the behavior when the values of the options were low, allowing exploration, but did not drive learning. Indeed, a model with only the biases (‘no value’) could not capture learning (Figures S1G and S1H, dark blue). In contrast, removing all biases had little effect on learning (‘value only’, Figure S1G, dark green) but decreased the quality of the fit (Figure S1H, dark green). Removing just the bias for the previous chosen color (but keeping other biases) had little effect on learning (‘no bias prev color’, Figure S1G) or the estimated attentional template (template for the Reset and ‘no bias prev color’ models were highly correlated, r=0.98/0.996, p < 0.001 for monkey B/S).

#### Comparison with alternative behavioral models

We compared the Reset model to two alternative models that did not include a template. First, in a ‘win-stay lose-forget’ (WSLF) model, subjects chose colors that were closest to the previously rewarded color. In other words, the animal’s attentional template was simply the previously rewarded color. After choosing a stimulus, if the received reward was above a fitted threshold then the chosen color would become the attentional template (‘stay’). If the received reward was below the threshold, then all values were set to 0 and the animal did not have an attentional template (‘forget’). The model included the same biases as the Reset model described above, except for the bias for the previous chosen color (as it was redundant, 8 parameters).

Second, a ‘win-stay lose-shift’ (WSLS) model was identical to the WSLF model with the exception that, after a below-threshold reward, the attentional template was taken to be the color that was opposite on the color wheel to the previously chosen color (‘shift’).

Both the WSLF and WSLS models propose a different mechanism for the template update, which is non-incremental and independent of the reward prediction error. Neither model fit monkeys’ behavior well (Figure S1G) and both models had a higher BIC than the selected Reset model (with 6 radial basis functions, see above, Figure S1H). This supports the conclusion that monkeys integrated information incrementally over trials to construct the attentional template.

Finally, we test a model with two learning rates, one for positive and one for negative reward prediction errors, was an extension of model with a reset and so it had 13 parameters. It was fitted as described above with 6 radial basis functions. Models with one and two learning rates were compared using BIC.

#### Validation of model fits

We validated our fitting and model selection procedure by generating choices based on the No Reset, Reset and WSLF models using trial conditions from both monkeys (i.e., the true template and the colors and locations of the stimuli, N=29,874 trials). In these models, the agent selects a target based on its value with the same softmax noise that was used for the animals’ models’ fit. We varied three parameters: the radial basis function concentration, the learning rate, and the reset threshold (or reward threshold for WSLF; see Table S3 for complete list of parameters and see Figure S1I *top left* for overall accuracy of No Reset and Reset models). For the reset threshold we tested values that matched the number of trials when a reset is detected in the monkey’s behavior (0.4% of trials overall for a reset threshold of 0.5, Figure S1I *top right*, thus matching our ability to detect resets in the model). For each model and set of parameters, we generated 10 sequences of choices that differed from one another only due to noise in the target selection process with the softmax. This led to a total of 140 generated sequences of choices.

We then fit the No Reset, Reset and WSLF models to the choices generated by each of the three generative models (thus fitting 420 models). We found that in all cases the model with the lowest BIC was the generative model, reflecting the fact that our model fitting accurately recovered the underlying model (all ΔBIC>14, Figure S1I *bottom left*, WSLF models are not shown in the figure but, all ΔBIC>621 compared to No Reset and Reset models). Additionally, we could correctly identify the reset threshold, particularly for the 0.5 value which best matched the statistics of the monkeys’ behavioral data fit (Figure S1I *bottom right*, green dots compared to the gray dotted line representing the proportion of resets in the data). When fitting the generative No Reset models with Reset models, the reset threshold was set to a high value reflecting how unlikely reset events were (Figure S1I *bottom* right, grey dots).

The goal of the behavioral models was to estimate the latent attentional template of the animal, as this formed the basis for the neural analyses. Therefore, we tested the ability of the fitting procedure to recover the generative attentional template. Across all Reset models, we recovered the estimated attentional template with high accuracy (circular correlation, reset threshold 0.3: all r>0.93, p < 0.001, black dots; reset threshold 0.5: all r>0.96, p<0.001, green dots; Figure S1J). This was also the case for the No reset models (all r>.94, p<0.001, grey dots, Figure S1J). This suggests that our model fitting procedure identified the template accurately across a range of parameters.

Finally, as noted above, because the learning rate and radial basis function concentration can trade-off, they are not uniquely recovered (although their ratio was recovered, particularly for models that match the statistics of monkeys’ behavior, Figure S1K, *top left*, green dots). However, fitting recovered the values of the biases (Figure S1K) and the preferred color (median absolute circular error = 0.10 rad, standard deviation = 0.09 rad).

Overall, our fitting procedure was able to recover the correct generative model, attentional templates, and parameters across all tested models.

#### Evaluation of the effect of value on choice behavior

To evaluate how the model-derived value of the options on screen affected choices, we used generalized logistic regressions (Matlab function ‘fitglm’, all regressors were z-scored). First, we looked at the effect of the value of the best (closest to the template), second best and worst options on accuracy (choosing the best option, not according to the behavioral model but the closest to the true template). In both monkeys, the greater the value of the best option, the highest the accuracy (all p<0.001), while the greater the values of the second best and worst option, the worst the accuracy (all p<0.001). We observed the same pattern when looking at the model’s probability to choose the true best option (using a linear regression, all p<0.001), which indicates that the model captures this feature of the decision-making process.

Second, we looked at the interaction between the distance to the estimated template of the best and second-best options (that had the greatest influence on the behavior) and the uncertainty about the template, captured by the entropy of the value function (Equation 15). Here, we used as regressor 1 - normalized absolute value of the distance, such that it would be equal to 1 if the target was the template and 0 if the target was at a distance of π from the template. We found that the closer the best target was to the template the greater the accuracy, but that this effect decreased with uncertainty (p<0.001). Furthermore, as noted above, the closer the second-best target was to the template the lower the animal’s accuracy, but this effect also decreased with uncertainty (p<0.001). Additionally, we found that the uncertainty generally decreased the accuracy and that when both the best and second-best were close to the template, accuracy decreased (all p<0.001). Again, we observed the same pattern when looking at the model’s probability to choose the true best option (using a linear regression, all p<0.001), which indicates that the also model captures this feature of the decision-making process.

Altogether, these results are consistent with the animal’s deliberating between stimuli based on their template-driven value.

#### Electrophysiological recordings and signal processing

Animals were implanted with a titanium headpost to immobilize the head and with two titanium chambers to provide access to the brain. The chambers were positioned using 3D models of the brain and skull obtained from structural MRI scans. Chambers were placed to allow for electrophysiological recording from LPFC, FEF (frontal chamber) and LIP (parietal chamber).

Epoxy coded tungsten electrodes (FHC Inc, Bowdoin, ME) were used for both recording and micro-stimulation. Electrodes were lowered using a custom-built micro-drive assembly that lowered electrodes in pairs from a single screw. Recordings were acute; up to 60 electrodes were lowered through intact dura at the beginning of each recording session and allowed to settle for 2–3 hours before recording. This enabled stable isolation of single units over the session. Broadband activity (sampling frequency = 30 kHz) was recorded from each electrode (Blackrock Microsystems, Salt Lake City, UT). We performed 8 recording sessions in Monkey B and 9 sessions in Monkey S.

Based on MRI scans, we identified electrodes that were likely to be located in the FEF for each monkey and confirmed our identification using electrical micro-stimulation. Based on previous work, we defined FEF sites as those for which electrical stimulation elicited a saccadic eye movement.^83^ Electrical stimulation was delivered in 200ms trains of anodal-leading bi-phasic pulses with a width of 400ms and an inter-pulse frequency of 330Hz. For monkey B, we found 4 sites that responded to electrical stimulation by evoking a saccade with a stereotyped eye movement vector at ≤100Ma (reliability of the stimulation was: 83% at 50 Ma, 67% at 50Ma, 50% at 100Ma, and 25% at 100Ma). For monkey S, we found 5 sites that responded to electrical stimulation (reliability of the stimulation was 67% at 50Ma, 57% at 50Ma 28% at 50Ma, 43% at 100Ma, 67% at 100Ma). Sites anterior and lateral to the FEF were labelled as LPFC.

Before the start of each session, monkeys performed a delayed match to sample task during which a target was presented for 125±25ms in 8 possible locations around a central fixation cross. After a delay, monkeys were instructed to saccade to the location of the sample. At the start of the session, this delay was short but rapidly increased to reach up to 650ms for monkey B and 600ms for monkey S. We identified pairs of electrodes located in LIP using the following criterion. We filtered the signal using a 4-pole 300–3000Hz band-pass Butterworth filter. We set the threshold for the detection of a spike by multiplying an estimate of the standard deviation of the background noise $\sigma_{n}$ by a factor β that we varied between 1.8 and 5^84^:

(Equation 16) $$\sigma_{n}=median\left(\frac{\left|x\right|}{0.6745}\right)$$

Time points at which the signal x crossed this threshold with a negative slope were identified as putative spiking events. Repeated threshold crossings within 32 samples (1.0667ms) were excluded. A channel was considered to be in LIP if there was a significant modulation of the spike rate during the delay (calculated by counting the total number of spikes during the delay) by the location of the remembered location of the sample in correct trials. We selected the value of β (= 2.2) that maximized the number of LIP channels detected using this method. Both electrodes in a pair were considered in LIP if at least one electrode reached this criterion.

Electrophysiological signals for the main task were filtered offline using a 4-pole 300Hz high-pass Butterworth filter. The spike detection threshold for all recordings was set equal to $-4\sigma_{n}$ (Equation 16). Repeated threshold crossings within 32 samples (1.0667ms) were excluded. Waveforms around each putative spike time were extracted and were manually sorted into single units, multi-unit activity, or noise using Plexon Offline Sorter (Plexon). The experimenter was blind to trials during all steps of preprocessing and spike sorting.

#### Single neuron sensitivity to the factors of the task and model selection

To estimate the sensitivity of single neurons to the factors of interest, we used a 10-fold cross validation procedure (Figure S3B). We fit the z-scored firing rates with the model representing the factors of interest using the Matlab function ‘fmincon’ with the default algorithm ‘interior-point’ that enables to solve a constrained minimization problem. To understand how neurons encoded task-relevant information, we fit four competing models for each individual neuron:

*Estimated template model*:

(Equation 17) $$FR(t)=\beta_{0}+\beta_{estimated\;template}.\frac{e^{\kappa .\text{cos}\left(estimated\;template(t)-\theta_{0}\right)}}{2\pi l_{0}(\kappa )}+e_{t}$$

Where $I_{o}$ is defined in Equation 2, *estimated template* is defined in Equation 13 and $e_{t}$ is the residual. We used the same model for the true template model (Figures S3F and S3G).

*Expected value model*:

(Equation 18) $$FR(t)=\beta_{0}+\beta_{EV}.\int_{-\pi }^{\pi }v(\theta ,t).\frac{e^{\kappa .\text{cos}\left(\theta -\theta_{0}\right)}}{2\pi I_{o}(\kappa )}d\theta +e_{t}$$

Where v(θ,n) is the value of the option color θ at trial n defined at Equation 4.

*Mean value model* (equivalent to Equation 18 with a κ close to 0):

(Equation 19) $$FR(t)=\beta_{0}+\beta_{mean\;value}.mean(v(\theta ,t))+e_{t}$$

*Chosen color model*:

(Equation 20) $$FR(t)=\beta_{0}+\beta_{chosen\;color}.\frac{e^{\kappa .\text{cos}\left(\theta_{chosen}(t)-\theta_{0}\right)}}{2\pi I_{o}(\kappa )}+e_{t}$$

We used the following constraints: $\beta_{0}\in [-10\;10],\beta_{model}\in [-1010],\theta_{0}\in [-2\pi \;2\pi ]$ and $\kappa =10^{\rho }$ with ρ∈[−2.52.5] and checked that all solutions converged. We computed the explained variance (${\text{R}}^{2}$) of the model on the validation trials. We only included neurons with more than 500 trials in our analysis. A neuron was considered significantly sensitive to the factor of interest in the model if the mean ${\text{R}}^{2}$ across validation sets was positive. Models were mutually exclusive such that if a neuron was significantly sensitive to several factors, the winning model was the one with the highest mean ${\text{R}}^{2}$. Except for the mean value model, all three other models assume that neurons have a smooth circular tuning for the variable they represent.

To estimate the significance of the proportion of neurons for each mutually exclusive model, we randomized the trial condition labels (500 shuffles) and computed the proportion of significant neurons for each model. We then compared the proportion of neurons for each model to this null distribution.

### Pseudo-population decoding and cross-template generalization

To understand how the neural population represented the task, we combined neurons recorded during different sessions to create 100 independent pseudo-populations of N neurons for our regions of interest. N was the minimum number of neurons across regions that had at least 60 trials for each condition (some populations had more). For each pseudo-population, the activity matrix was a concatenation of the activity of N neurons (randomly selected with replacement) in m randomly selected trials in each condition. N and m were the same for all regions and across all time points and locations (when relevant) but specific to each analysis (estimated template, choice, chosen value, target color and chosen color).

For the estimated template (Figures 2C–2E), the true template (Figure S3G), stimulus color (Figure S5B) analyses, we trained three classifiers: one for each color bin to discriminate the pseudo-population response at each time point, such that each classifier hyperplane separated the ‘in color bin’ vs. ‘out of color bin’ (with a randomly subsampled equal number of trials for each of the two other bins). For the estimated template and true template analyses, trials were balanced across progression in the block (1/3, 2/3, 3/3), yielding 9 conditions with an equal number of trials (‘pink’, ‘brown’ and ‘blue’ templates x 3 levels of progression in the block). In our task, the location and color of the stimuli was random, such that by design, there was no relationship between the stimulus color and value at a location and the color of the template. For the stimulus color analysis, trials were balanced across chosen and unchosen stimuli (Figure S5B), yielding 6 conditions with an equal number of trials (‘pink’, ‘brown’ and ‘blue’ stimulus color x chosen and unchosen). For the choice (Figures 5E, S5D, and S5E), chosen color (Figures 5D and S5C) and chosen value (Figures 5B and S5A), we trained one classifier. For the choice and chosen value analyses, trials were balanced across estimated template color with three bins, yielding 6 conditions with an equal number of trials (chosen/unchosen or low/high chosen value (median split) x ‘pink’, ‘brown’ and ‘blue’ templates). For the choice analysis, we used the same number of trials for all locations. For the chosen color analysis, trials were balanced across estimated template color but with only two bins, yielding 4 conditions with an equal number of trials (‘pink’ and ‘green’ chosen color x ‘pink’ and ‘green’ templates). Note that chosen color and chosen value tended to be correlated when the template and chosen color bins were the same (e.g., pink chosen colors tend to have a high value when pink is the template), while they tended to be inversely correlated when the bins were different (e.g., pink chosen colors tend to have a low value when green is the template). For the choice location for each display analyses (Figure S5F), we trained three classifiers (one for each location), yielding 3 conditions with an equal number of trials. We used the same number of trials for all four displays. In all cases, a subset (20%) of trials were withheld for validation, again balanced across conditions.

For all analyses, we used the same number of neurons and trials for all three regions. For the estimated template analysis (Figures 2C–2E), the pseudo-population was made of 100 neurons, and decoders were trained/tested on 80/20% of 61 trials per progression level (three levels) and estimated template color bin (three bins). For the chosen value analysis (Figure 5B), the pseudo population was made of 110 neurons, and the decoders were trained/tested on 80/20% on 128 trials per estimated template bin, three bins (with a balanced number of low/high chosen value trials). For the chosen color (Figure 5D), it was made of 133 neurons, trained/tested on 80/20% of 160 trials per estimated template bin, two bins (with a balanced number of trials for each chosen color bin (2 bins)). For the choice analysis at each location (Figure 5E), we used 90 neurons and the decoders were trained/tested on 80/20% on 120 trials per estimated template bin, three bins (with a balanced number of chosen and unchosen trials).

The goal of the cross-template generalization analysis (Figure 5) was to establish whether the neurons in each region represented the value of the chosen stimulus or the color of the (chosen) stimulus in a manner that generalized across templates. First, for the chosen value, we tested whether a decoder trained across three template colors (with an equal number of trials for each bin of color) could decode the chosen value. It could (Figure S5A). This suggests there is a common representation of the chosen value across templates. We also tested whether a chosen value decoder trained on one template color generalized to other template colors. Again, it could (Figure 5B). Finally, we showed that neurons specifically represented the chosen value by fitting a parametric linear model trained on all template colors that controls for the reward (Figure 5C). All analyses show chosen value is represented in a stable manner across template colors.

Second, for the color of the stimulus, we tested whether the neurons represented the color of the stimulus at a given location (using the exact same decoding method as for the template color). Stimulus color could not be decoded (Figure S5B). Second, we tested whether the chosen color was represented when controlling for the template color (as the two are correlated) and again found that it was not the case (Figure S5C). This was true for both the population (using a binary classifier) and for individual neurons (Figures S3B and S3C). As we could decode template color using a binary classifier (Figure S5C), we do not think the inability to decode color reflects a limitation of the decoding approach. Finally, we found chosen color could be decoded within a template (Figure 5D). Indeed, within a template, the chosen color is highly correlated with the value, and so value representations can be used to decode ‘color’. Again, this result is consistent with a generalized value representation. We also found that the choice decoder generalized across templates (Figure 5E), in line with a fixed read-out for the decision-making process across templates.

We used a standard linear support vector machine (SVM) classifier fit with the Matlab function ‘fitcsvm’ with the default solver ‘Sequential Minimal Optimization’. To minimize overfitting of the classifiers, fitcsvm uses the L1 norm for regularization (with box constraint as hyperparameter) and a kernel scaling parameter. The hyperparameters were set to minimize the 10-fold cross-validation loss. We used the default optimizer (‘bayesopt’) and the acquisition function ‘expected-improvement-plus’ that prevents the over-exploitation of one region of the parameter landscape. The projection of the trial input (the vector of the firing rates of the N neurons) onto the hyperplane defined the degree to which this trial is in or out of the class also called the score. We transformed this score in the posterior probability to belong to the class using the Matlab function ‘fitPosterior’.

The estimated template (Figures 2C–2E), the true template (Figure S3G), and stimulus color (Figure S5B) analyses used three (or four) classifiers and so we used two ways of calculating the overall classification accuracy. First, we took the class with the maximum posterior probability as the class for a particular trial. Second, we trained a feedforward, fully connected neural network for classification (function ‘fitcnet’ in MATLAB with default settings) on the posterior probability across all three classes (withholding validation trials). The latter method tended to improve the classification accuracy and could account for distortions in the representation of the color wheel, so we report the results of this method. Classification accuracy was taken as the probability for a trial to be correctly classified on validation trials (chance level was 1/3 or 1/4). For all other analysis, that had only one classifier, we used the direct output of the SVM classification. Here as well, classification accuracy was taken as accuracy on validation trials (chance level was 1/2). For the choice location classifier (Figure S5F), we took the class with the maximum posterior probability as the class for a particular trial.

One concern might be that drift in neural responses over time lead to artifactual template information. To ensure this was not the case, templates were randomly chosen over time. Furthermore, when decoding the estimated template, there were on average 3.5/3.7/3.6 blocks of template in each bin for LIP/FEF/LPFC. However, a few neurons included only a template from 1 block in a bin of the estimated template. Removing these neurons (i.e., the same analysis shown in Figure 2D ‘across’), ensuring all bins included at least two different blocks of time, did not affect the decoding accuracy.

Statistical significance was estimated with one-sided z-tests against chance level (1/3 or 1/2 for one classifier) with a Bonferroni correction for multiple comparison for time series.


(Equation 21) $$p=1-normcdf\left(\frac{\overset{‾}{y}-\mu }{\sigma_{y}}\right)$$

Where $\overset{‾}{y}$ is the sample mean, $\sigma_{y}$ is its standard deviation, μ is the hypothesized population mean and ‘normcdf’ is the function that returns the cumulative distribution of the standard normal distribution (computed with the Matlab function ‘normcdf’). Note that we used a z-test rather than a t-test because the sample size is defined arbitrarily by the number of bootstraps.

Cross-template generalization was assessed by training the SVM classifier using trials from one color bin of the estimated template and computing the classification accuracy either on validation trials from the same estimated template bin (‘within’) or from the other template bins (‘across’) (Figures 5 and S5). Statistical significance was estimated with paired one-sided z tests (‘within-across’) against 0 using the mean and standard deviation of the bootstrap distributions (Equation 21) and with a Bonferroni correction for multiple comparison for time series.

#### Principal component analysis

For the Principal Component Analysis in Figure 2 (PCA; Figures 2E and S3D), trials were sorted into 3 estimated template color bins and 20 time points (200ms windows centered on the onset of the targets), yielding 60 total conditions. The activity matrix X was the mean population activity (across trials) for each condition and each neuron. The principal components of this matrix were identified by decomposing the covariance matrix C of X using the Matlab function ‘pca’:

(Equation 22) $$C=PDP^{T}$$

where each column of P is an eigenvector and D is a diagonal matrix of corresponding eigenvalues of C. The first 4 eigenvectors (in decreasing order of variance explained) respectively explained 95% /89% /90% of the variance in LIP/FEF/LPFC. We constructed a reduced 3-dimensional space using the first, second and fourth eigenvectors (Figure 2E) or the first three eigenvectors (Figure S3D) and projected the population activity vector for a given condition into this reduced dimensionality space. The percent explained variance and effective dimensionality were estimated with 100 bootstraps, by drawing with replacement from the 146 /216 /475 neurons in LIP/FEF/LPFC. The effective dimensionality was computed as:

(Equation 23) $$\text{dim}=\frac{{\left(\sum_{i=1}^{M}\gamma_{i}\right)}^{2}}{\sum_{i=1}^{M}\gamma_{i}^{2}}$$

With M=59 as the degrees of freedom.

For the PCA in Figure S5 (Figure S5G), trials were sorted into 3 estimated template color bins, 2 bins for chosen color value (median split) and 25 time points (200ms windows centered on the onset of the targets and starting at −400ms), yielding 150 total conditions. Here, we removed the influence of time by subtracting the average activity across condition for each time point and neuron. The first 3 eigenvectors (in decreasing order of variance explained) respectively explained 68/74/60% of the variance in LIP/FEF/LPFC.

#### Multidimensional scaling analysis

For the Multidimensional scaling Analysis in Figure 4, trials were sorted into 6 estimated template color bins. The activity matrix X was the mean population activity (across trials) for each bin and for each neuron in a −600 to 300ms window centered on targets onset. First, we used the Matlab function ‘pdist’ to obtain the dissimilarity matrix of X, the pair-wise Euclidean distance between the estimated template bins in the N dimensional space, N being the number of neurons. Then we used the Matlab function ‘mdscale’ to recreate the data in 2 dimensions.

#### Trial-by-trial estimated template decoding using population decoding

We used a decoding approach to estimate the trial-by-trial neural representation of the estimated template. These analyses were done independently for each session, on simultaneously recorded neurons. For each block, we isolated the first 35 trials of the block and labelled them as validation trials that we did not use to train the classifiers nor the neural network. We trained three classifiers, one for each color bin, on balanced trials across color bins as above. Then, we trained a feedforward, fully connected neural network that took the posterior probability of each classifier as input and output the predicted cosine and sine of the estimated template (using the Matlab function ‘feedforwardnet’; two layer network; hidden layer of size 10 using the *relu* activation function, ‘poslin’ in Matlab; output layer of size 2 with a linear activation function). Thus, on each trial n, we could estimate the color θ(n) of the neural estimated template. Note, while the relative angular distance between colors is defined by color space, the absolute angular value of the color wheel was arbitrarily chosen such that ‘red’ was defined as 0. Decoding accuracy was calculated on validation trials by calculating the circular distance (Equation 11) between the estimated template (from the behavioral model) and neurally predicted estimated template.

The update was computed such that it should always be positive if it was i) toward the chosen color for a positive RPE and ii) away from the chosen color for a negative RPE:

(Equation 24) Update(n)=sign(RPE(n−1)).sign(d(ET(n−1),CC(n−1))).d(ET(n−1),ET(n)).

Where d is the angular distance (Equation 11) and sign is the function that takes the value 1 if its argument is positive and −1 if it is negative. ET is the estimated template (either from the behavioral model or decoded from the neural population) and CC is the chosen color. We tested whether this update and the circular mean across updates in a session tended to be positive (using a T-test), which would indicate that the update tended to be in the direction predicted by the model.

Significance of the decoding was assessed by comparing the observed mean circular distance between the decoded and estimated template to what would be expected by chance. Chance distributions were estimated using a permutation approach (randomizing the template condition labels 1000 times and calculating the null distribution of the mean circular distance). For each session, the z-score of the observed difference was estimated by subtracting the mean of the null distribution and normalizing by the standard deviation of the null distribution. The resulting distribution of z-scored mean circular distances across sessions was then tested against 0 (Figures 3B/S4B and 3D/S4B). We used the same approach to test for significance of the distances of the distance between two estimated templates and two decoded templates (Figure 3D/S4C) and the distance between the update toward from the chosen color after positive reward prediction errors between the estimated and behavioral templates (Figure 4B). We used the same approach across all trials (Figure S4A). In this case, the p-value was computed by directly comparing the raw observed difference to the null distribution. Circular correlations were computed using the MATLAB circular statistics (RRID:SCR_016651) toolbox.

As noted in the legend for Figure 3D, we observed a slight bias in the neural decoding of the color template which is reflected in the fact that the correlation between the neural and behavioral templates (shown in the thick dashed line) lies below the identity line (i.e., slope < 1). This bias reflects the fact that, in our task, behaviorally estimated attentional templates were slightly biased towards certain colors (indicated as ‘pi’ in Figure 3D). While this could be due to random chance, it could also reflect biases in the representation of color. Previous work has shown behavior is biased towards certain colors, both in perception^85^ and in working memory.^45^ As our task involves both perceiving colors and maintaining an attentional template for color in working memory, these biases could influence monkey’s behavior in our task. The neurally estimated template matches the behavioral templates, leading to the bias observed in Figure 3D. Re-running the analysis on a subset of behavioral templates, such that the distribution of behavioral templates was more uniform, reduced the bias in the neural templates. Importantly, this did not change our results – the same degree of circular correlation between the neural and behavioral templates was observed (r = 0.3, p<0.001).

#### Generalized multiple linear regressions and representation alignments

For the generalized linear regressions used in Figures 5, 6 and S6, we used the Matlab function ‘fitglm’. To remove any carry-over effect from previous trial, we removed the baseline firing rate (taken between −600ms and −300ms from targets onset) from the firing rate in each 200ms window. The firing rates were z-scored for each window.

For the ‘global’ regression, we used all trials (Figures 5, 6, and S6). The regressors were the reward magnitude, the value of the chosen stimulus, and the value of the unchosen stimulus with the highest value, both on the current trial and the previous trial. We also included the mean value of the current trial (taken as the mean value across all presented stimuli). For the ‘local’ value, the regressors were whether the stimulus was chosen, its value on chosen trials (0 otherwise), and its value on unchosen trials (0 otherwise). We only considered neurons where more than 500 trials were recorded, and with a firing rate that had enough variance to fit all the regressors.

At each time point, we defined the representation of a task factor (reward, chosen value and unchosen value) as the vector of the regression weights for this factor across neurons. We concatenated the distribution for the contralateral options (top right and bottom right on the screen) and the ipsilateral options (top left and bottom left on the screen) such that the distribution at each time point was 2 x N, with N as the number of neurons. The alignment r of these representations was taken as the Pearson correlation between the vectors (calculated using the Matlab function ‘corr’):

(Equation 25) $$r_{X,Y}=\frac{cov(X,Y)}{\sigma_{X}\sigma_{Y}}$$

Wherecov is the covariance and σ is the standard deviation. X and Y are the regressors estimated for each neuron on two different halves of the session. We performed 10 random splits of our sessions and estimated the mean correlation between the representations between the two sessions halves. To estimate the variability, we ran 5000 bootstraps, sampling N (global) or 2 x N neurons (local, the dimension of the vector, as above a concatenation of the two contralateral locations) with replacement.

We used a similar method to compare locations ‘in’ and ‘out’ of the receptive field. To estimate whether a location on the screen was ‘in’ the receptive field, we took advantage of the fact that we only presented stimuli in 3 of the 4 locations, so we could look if the presence or absence of a stimulus at a given location affected the firing rate of neurons. To do so, we used 4 independent linear models (fitted using ‘fitglm’, the ‘stim on’ regressor and an intercept) and assumed that a location was in the receptive field of a neuron if there was a significant modulation by the stimulus presence (p<0.005 for the ‘stim on’ regressor, in a 50 – 250ms from target onset, note that several locations could be in a neuron’s receptive field). We then concatenated the distribution for the ‘in’ options and the ‘out’ options (subsampled to match the number of ‘in’ options).

We first estimated the split-half reliability of the representations by comparing the representation of the same factors computed in the two halves of the session (Figures 5C, 6A–6D and S6A–S6E). This gives us access to the maximal alignment that could be found between the representations. Statistical significance was estimated using a paired one-sided ztest against 0 with a Bonferroni correction for multiple comparisons across time. Comparison between strengths of reliability was estimated with a paired two-tailed z-test against 0.


(Equation 26) $$p=2.\left(1-normcdf\left(\left|\frac{\overset{‾}{y}-\mu }{\sigma_{y}}\right|\right)\right)$$

With the same variables as defined in Equation 21.

When looking at the alignment between two different representations (either two different regressors or the same regressors at different locations), we only compared the representation in two different halves of the session such that the same trials were never used to compute the compared representations (Figures 6C, 6D, S6E, and S6G). To estimate the degree of alignment between representations, we computed the correlation disattenuation^54^:

(Equation 27) $$\gamma =\frac{r_{X,Y}}{\sqrt{{r_{X,X}}^{2}.{r_{Y,Y}}^{2}}}$$

Where $r_{X,Y}$ is the split-half correlation between the representation of the factors X and Y defined in Equation 25, $r_{X,X}$ and $r_{Y,Y}$ are the split-half reliability of the representations X and Y respectively defined above. Statistical dissimilarly was estimated using a one-sided ztest against 0 with a Bonferroni correction for multiple comparisons across time points (22 time points).

To estimate the overall alignment across locations, we computed the area under the curve of the representation reliability (distance index = 0) or alignment across locations over time (22 time points, Figure S6E) and over bootstraps (Figures 6C and S6G). We also looked at the ‘early’ period (Figure S6L, up to 300ms from target onset in Figure S6H). Statistical significance was estimated using a paired one-sided z-test (assuming that smaller distances would be more similar) against 0 with a Bonferroni correction for multiple comparisons (6 pairs).

To compute the z-scored explained variance of the chosen value representation across locations we used the split-half procedure described above. We estimated the chosen value regressor in one half of the session (either for the same of another location) and computed the ${\text{R}}^{2}$ of the regression in the other half of the session. We performed 250 shuffles of the chosen value label across trials and z-scored the ${\text{R}}^{2}$ with the shuffled ${\text{R}}^{2}$ distribution for each neuron. Statistical significance was estimated using a one-sided z-test against 0 across neurons. To estimate the overall explained variance across locations (Figure S6F), we computed the area under the curve of the explained variance across time (22 time points, Figure S6H) across neurons. Statistical significance was estimated using a paired one-sided z-test (assuming that smaller distances would be more similar) against 0 with a Bonferroni correction for multiple comparisons (6 pairs).

The peak of the unchosen value representation was computed by finding the time point at which the unchosen value reliability was maximal for each bootstrap. Statistical comparison between brain regions was estimated using a two-sample two-tailed z-test.


(Equation 28) $$p=2.\left(1-normcdf\left(\left|\frac{\overset{‾}{x}-\overset{‾}{y}}{\sqrt{\sigma_{x}^{2}.\sigma_{y}^{2}}}\right|\right)\right)$$

With the same variables as defined in Equation 21.

### QUANTIFICATION AND STATISTICAL ANALYSIS

All data were presented as means ± SEM, unless otherwise indicated. The statistical analyses performed were indicated in the main text and detailed in methods. Statistical comparisons were analyzed in Matlab (Mathworks), and included parametric statistics, circular statistics, and non-parametric, permutation-based statistics, as detailed in STAR Methods. Unless otherwise indicated, all statistics were corrected for multiple comparisons. Figures were prepared with Matlab.

## Supplementary Material

Supplemental Tables

1

## Figures and Tables

**Table:** KEY RESOURCES TABLE

REAGENT or RESOURCE	SOURCE	IDENTIFIER

Deposited data		

All neural data and code supporting figures.	This paper	Zenodo: https://doi.org/10.5281/zenodo.10529801
Behavioral and neural data.	This paper	Zenodo: https://doi.org/10.5281/zenodo.10529801

Software and algorithms		

Analysis code.	This paper	Zenodo: https://doi.org/10.5281/zenodo.10529801
MATLAB	Mathworks	RRID:SCR_001622
VBMC toolbox	Acerbi^82,81^	https://github.com/lacerbi/vbmc
Plexon Offline sorter	Plexon	https://plexon.com/products/offline-sorter/
circular statistics	Mathworks	RRID:SCR_016651
Violin plot	Bechtold and Fletcher (2021)^38^	https://github.com/bastibe/Violinplot-Matlab

## Highlights

- Monkeys repeatedly learned new attentional templates to select visual stimuli
- Neural representations of templates were structured in the prefrontal and parietal cortex
- Attentional templates were iteratively updated after each trial based on reward feedback
- Sensory inputs were translated into value, allowing attention to generalize across tasks
