# Supplementary material for: Learning attentional templates for value-based decision-making
Source: Cell. Author manuscript; Available in PMC 2025 Mar 14. (PMC11574977; doi:10.1016/j.cell.2024.01.041)
Supplement: Supplemental Tables [file NIHMS2030175-supplement-Supplemental_Tables.pdf]

<table>
<tr><th>Parameter</th><th>Initialization BADS (X_0)</th><th>Lower bound BADS</th><th>Upper bound BADS</th><th>Initialization VBMC1 (X_0)</th><th>Lower bound VBMC</th><th>Upper bound VBMC</th><th>Initialization VBMC n (X_0)</th></tr>
<tr><td>Concentration (κ)</td><td>2.5</td><td>0.5</td><td>5</td><td rowspan="12">BADS estimates</td><td>X_0-1 (>0.25)</td><td rowspan="5">X_0+1</td><td rowspan="12">VBMC(n-1) estimates</td></tr>
<tr><td>Learning rate (α)</td><td>0.5</td><td>0</td><td>6</td><td rowspan="3">X_0-1 (>0)</td></tr>
<tr><td>Volatility*</td><td>0.05</td><td>0</td><td>1</td></tr>
<tr><td>Reset threshold$_0$*</td><td>0.7</td><td>0</td><td>1.2</td></tr>
<tr><td>Bias color preference</td><td>0</td><td>0</td><td>1</td><td>X_0-1</td></tr>
<tr><td>Preferred color</td><td>0</td><td>-2π</td><td>2π</td><td>X_0-π</td><td>X_0+π</td></tr>
<tr><td>Bias previous color</td><td>0</td><td>-1</td><td>1</td><td rowspan="6">X_0-1</td><td rowspan="6">X_0+1</td></tr>
<tr><td>Bias location 2</td><td rowspan="5">0</td><td rowspan="5">-1</td><td rowspan="5">1</td></tr>
<tr><td>Bias location 3</td></tr>
<tr><td>Bias location 4</td></tr>
<tr><td>Bias bigger stim</td></tr>
<tr><td>Bias smaller stim</td></tr>
</table>

Table S1: Priors for fitting model parameters, related to STAR Methods. Table includes the priors for the difference steps used when fitting the behavioral model. See “Behavioral model fit and selection” in STAR Methods for details. * indicates parameters used for “Reset” model only.

Parameter	Monkey B	Monkey S
Concentration (κ)	1.6411 ± 0.0216	1.8154 ± 0.0331
Learning rate (α)	0.4858 ± 0.0103	0.2832 ± 0.0077
Volatility	0.5121 ± 0.2322	0.406 ± 0.0020
Reset threshold$_0$	0.8751 ± 0.0013	0.6979 ± 0.0081
Bias color preference	0.0554 ± 0.0157	0.1956 ± 0.0082
Preferred color (rad)	-1.6218 ± 0.2767	-2.7138 ± 0.0899
Bias previous color	0.4183 ± 0.0170	0.4619 ± 0.0094
Bias location 1 (fixed)	0	0
Bias location 2	0.1236 ± 0.0095	0.0574 ± 0.0069
Bias location 3	0.0557 ± 0.0126	0.0207 ± 0.0069
Bias location 4	-0.0216 ± 0.0103	0.0811 ± 0.0070
Bias bigger stimulus	0.1342 ± 0.0241	-0.0487 ± 0.0456
Bias smaller stimulus	-0.8133 ± 0.0430	0.1 ± 0.0402

Table S2: Model parameter fits for each monkey, related to STAR Methods. Mean ± standard error across 300,000 samples from the parameters’ posterior likelihood distribution for the Reset model with 6 channels. For the preferred color, we computed the parameter estimate as the mean angular distance and the standard error to the mean using the angular distance to the mean (see equation 11 in STAR Methods).

Model	No reset				Reset (defined by Reset threshold$_0$)				WSLF	
Model #	1	2	3	4	1	2	3	4	1	2
Concentration (κ)	1		1.7		1		1.7		n/a	
Learning rate (α)	0.5	1	0.5	1	0.5	1	0.5	1	n/a	
Volatility	n/a				0.5				n/a	
Bias color preference	0.05									
Preferred color (rad)	-π/2									
Bias previous color	0.5								n/a	
Bias location 1 (fixed)	0									
Bias location 2	0.1									
Bias location 3	0.05									
Bias location 4	-0.02									
Bias bigger stimulus	0.1									
Bias smaller stimulus	-0.8									
Reward threshold	n/a								0	0.2

Table S3: Generative parameters for evaluating fitting procedure, related to STAR Methods and Figure S1. Table includes the mode parameters (rows) used when creating generative models for validating the fitting procedure. Models fell into three classes (columns) – No Reset, Reset, and Win-Stay, Lose-Forget (WSLF). Within each class, we tested several parameters, indicated by the different model numbers.
