# Supplementary material for: Learning attentional templates for value-based decision-making
Source: Cell. Author manuscript; Available in PMC 2025 Mar 14. (PMC11574977; doi:10.1016/j.cell.2024.01.041)
Supplement: 1 [file NIHMS2030175-supplement-1.pdf]

Supplemental figures

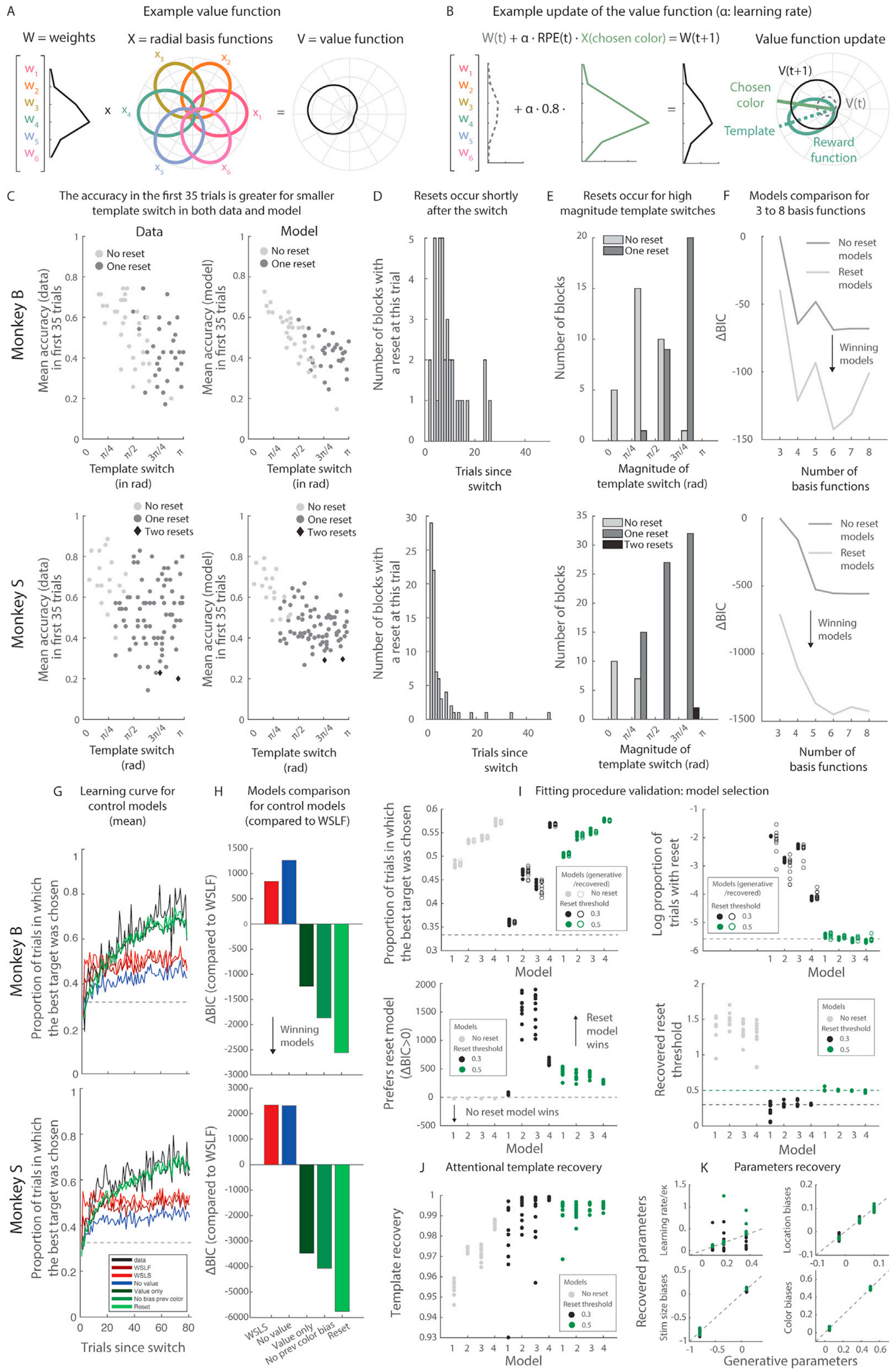

(legend on next page)

Figure S1. Extended behavioral analysis and model validation, related to Figure 1

(A) Example value function. On each trial, the value function is computed by combining the weights (left) and the radial basis functions (middle) centered on equally distanced colors to calculate the value function across colors (right).

(B) Example update of the value function. After a positive reward prediction error (RPE), here 0.8 (a very large RPE), the model weights on the radial basis functions increase as a function of their distance to the chosen color (here green). This leads to an increased updated value function for colors closer to the chosen color.

(C) Mean accuracy in the first 35 trials after a template switch for the subjects (left) and the model (right). The accuracy is greater when the previous and current templates are closer. Dot color and shape indicate whether a switch was detected in the model, this tends to occur for larger template switches. The model also captured the relationship between the magnitude of the change of template color and the monkey's performance ($r = -0.5455$, $p < 0.001$ for the data and $r = -0.6943$, $p < 0.001$ for the model, 61 template switches/$r = -0.2744$, $p = 0.0078$ for the data and $r = -0.5740$, $p < 0.001$, 93 template switches, for monkey B/S)

(D) Histogram of when resets are detected after the template switch for monkey B (top) and monkey S (bottom).

(E) Histogram of how many resets were detected per bin of magnitude of template switch (absolute value of the angular distance between the previous and current templates).

(F) Bayesian information criterion (BIC) of the "reset" and "no reset" models for 3 to 8 radial basis functions.

(G) Learning curves for monkey B (top, 69 blocks) and monkey S (bottom, 102 blocks). The colored lines represent the model's mean probability of choosing the true best target. Only models with a value function derived from the Q-learning with function approximation (shades of green) could capture learning. WSLF, win-stay lose-forget; WSLS, win-stay lose-shift. See STAR Methods for details of models. Because there are 3 targets, chance level is 1/3.

(H) BIC of the models compared with the win-stay lose-forget model.

(I) Validation of fitting procedure. Top left: overall model accuracy for the generative model (full dots) and the recovered model (empty dots) across various parameters (model #) and sampling (10 samples for each model, variability is due to the softmax noise in the target selection). Top right: log proportion of trials with a reset. Models with a reset threshold of 0.5 (green dots) best approximate the proportion of reset events in the data (gray dotted line). The model recovery approximates well the number of resets detected (compare full and empty dots for each model). Bottom left: model recovery: BIC was estimated for each generative model and is lowest for the correct generative model. Bottom right: the fitting procedure recovers the reset threshold. It has a very high value when the generative model is a no reset model because very few or no resets are detected.

(J) Estimated template recovery (circular correlation between the generative and fitted estimated attentional template, all $p < 0.001$).

(K) Parameter recovery for the ratio between the learning rate and the exponential of the radial basis concentration (κ) (top left), the three fitted location biases per model (top right), the stim size biases (bottom left), and the previous chosen color and preferred color biases (bottom right).

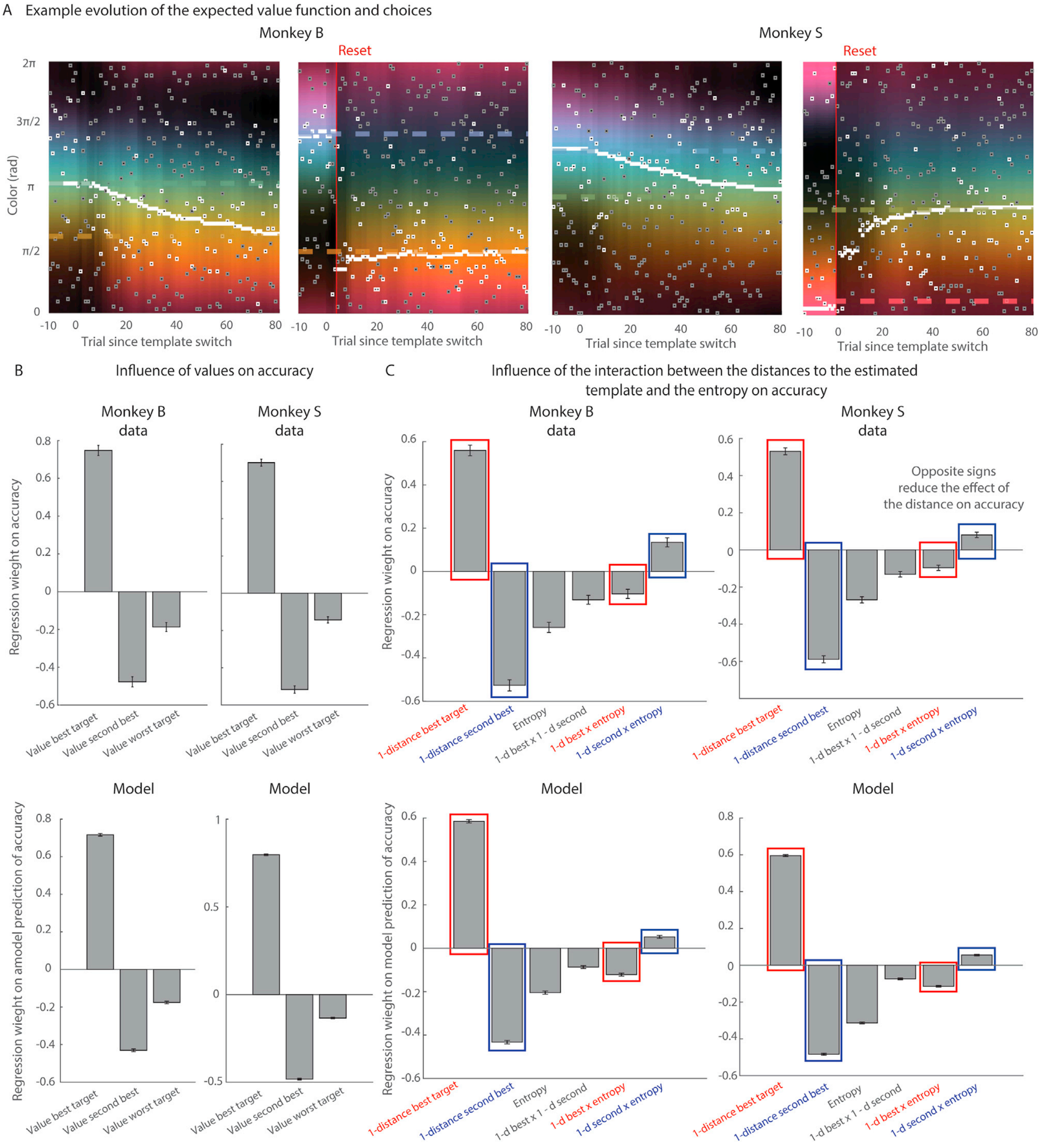

Figure S2. Choice behavior is captured by the model, related to Figure 1

(A) Examples of evolution of the expected value function (as in Figure 1G), chosen colors (white open squares) and unchosen colors (gray open squares) on each trial. The estimated template is indicated with a white full marker on each trial. The brighter the color, the higher the expected value according to the model. Previous and current templates are indicated with dashed lines. Chosen colors cluster around high values close to the estimated templates.

(B) Top: logistic regression weights of the value of the best, second-best, and worst option on accuracy. Bottom: linear regressions weights of the value of the best, second-best, and worst option on model derived probability to choose the true best option (equivalent of accuracy). Intercepts are not shown. All regressors were *Z* scored. Error bars represent standard error.

(C) Same as (B), but with the reversed normalized distance of the best and second-best option to the estimated template, the expected value entropy, and their interactions.

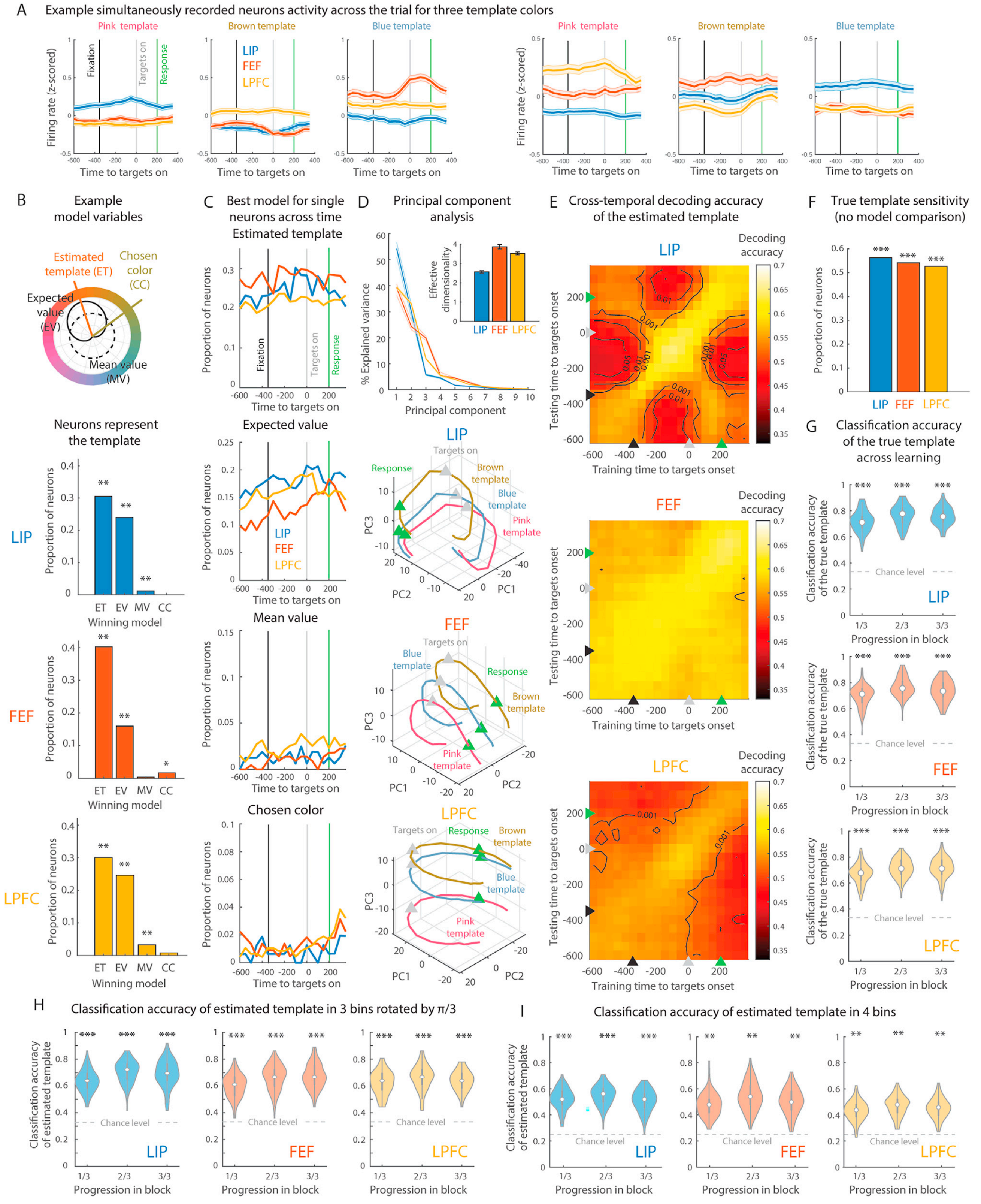

(legend on next page)

Figure S3. Extended analysis of the estimated attentional template representation, related to Figure 2

(A) Firing rate over time for three example, simultaneously recorded, neurons for three different bins of estimated template color (*z* scored over the full session). The ‘‘tuning’’ of the neuron was maintained over time in the trial.

(B) Top panel shows an example trial showing the difference between the estimated template (orange), the chosen color (green), the expected value function (black), and the mean value (dashed black). Bottom panel shows the proportion of significant neurons for the 4 mutually exclusive models: estimated template (ET), expected value (EV), mean value (MV), and chosen color (CC). Models were fit to 167/231/492 neurons in LIP/FEF/LPFC in a -600- to 300-ms window around the onset of the targets. The estimated template best explained neural activity in the largest group of neurons in all three regions (30.54/40.26/30.08% in LIP/FEF/LPFC, all $p \leq 0.002$, permutation test), although the expected value distribution was also well represented (29.95/16.02/24.59% of neurons in LIP/FEF/LPFC, all $p \leq 0.002$). Mean value and chosen color were comparatively less represented in all three regions (MV: 1.20%/0.92%/3.25% in LIP/FEF/LPFC, p = 0.008/0.0978/0.002, and CC: 0/1.73/0.23% in LIP/FEF/LPFC, p = 0.6447/0.01/0.1138). See STAR Methods for details on model comparison.

(C) Proportion of neurons significantly encoding the four mutually exclusive models in each region over time, relative to the onset of the targets (300 ms windows, as in Figure 2D).

(D) Top panel shows percent explained variance by the first 10 principal components (100 bootstraps, mean and 95% confidence interval). Inset shows the effective dimensionality (100 bootstraps, mean and standard error to the mean). Bottom panels show neural activity in all three regions projected into a reduced dimensionality space consisting of the first three principle components (PCs, in decreasing order of explained variance). LIP, 146 neurons; FEF, 216 neurons; LPFC, 475 neurons. The gray triangle represents the onset of the targets, and the green triangle represents the approximate time of response (200 ms after the onset of the targets). The projection along the 4th PC shows the separation of the three estimated templates’ color in a triangle shape with a linear effect of time. The projection in the first 3 PCs leads to a more cyclic effect of time.

(E) Cross-temporal decoding of the attentional template (as in Figure 2F, trained across all progression levels). x axis corresponds to time window used to train classifier, and y axis corresponds to the time window used for testing the classifier (on withheld trials). Color code indicates classification accuracy of template, and lines indicate significance level (z test on 100 bootstraps). Triangles represent the approximate time of fixation (black), targets onset (gray), and response (green).

(F) Proportion of neurons significantly sensitive to the true attentional template (i.e., the color used by the behavioral task). Thus, the representation of the template did not depend on the behavioral model.

(G) True template classification accuracy for each third of the block, estimated on withheld trials (as in Figure 2D, left half, 113 neurons per region, trained/tested on 80%/20% of 76 trials per progression level [three levels] and estimated template color bin [three bins]). Violin plot: central white dot is the median, thick vertical gray bar represents the 25th to 75th quartile, and area represents the kernel density estimate of the data.

(H) Same as Figure 2D, left half, but with bins rotated by $\pi/3$, 120 neurons per region, trained/tested on 80%/20% of 62 trials per progression level (three levels) and estimated template color bin (three bins).

(I) Same as Figure 2D, left half, but with 4 bins, 49 neurons per region, trained/tested on 80%/20% of 61 neurons. For all panels, *$p \leq 0.05$, **$p \leq 0.01$, ***$p \leq 0.001$.

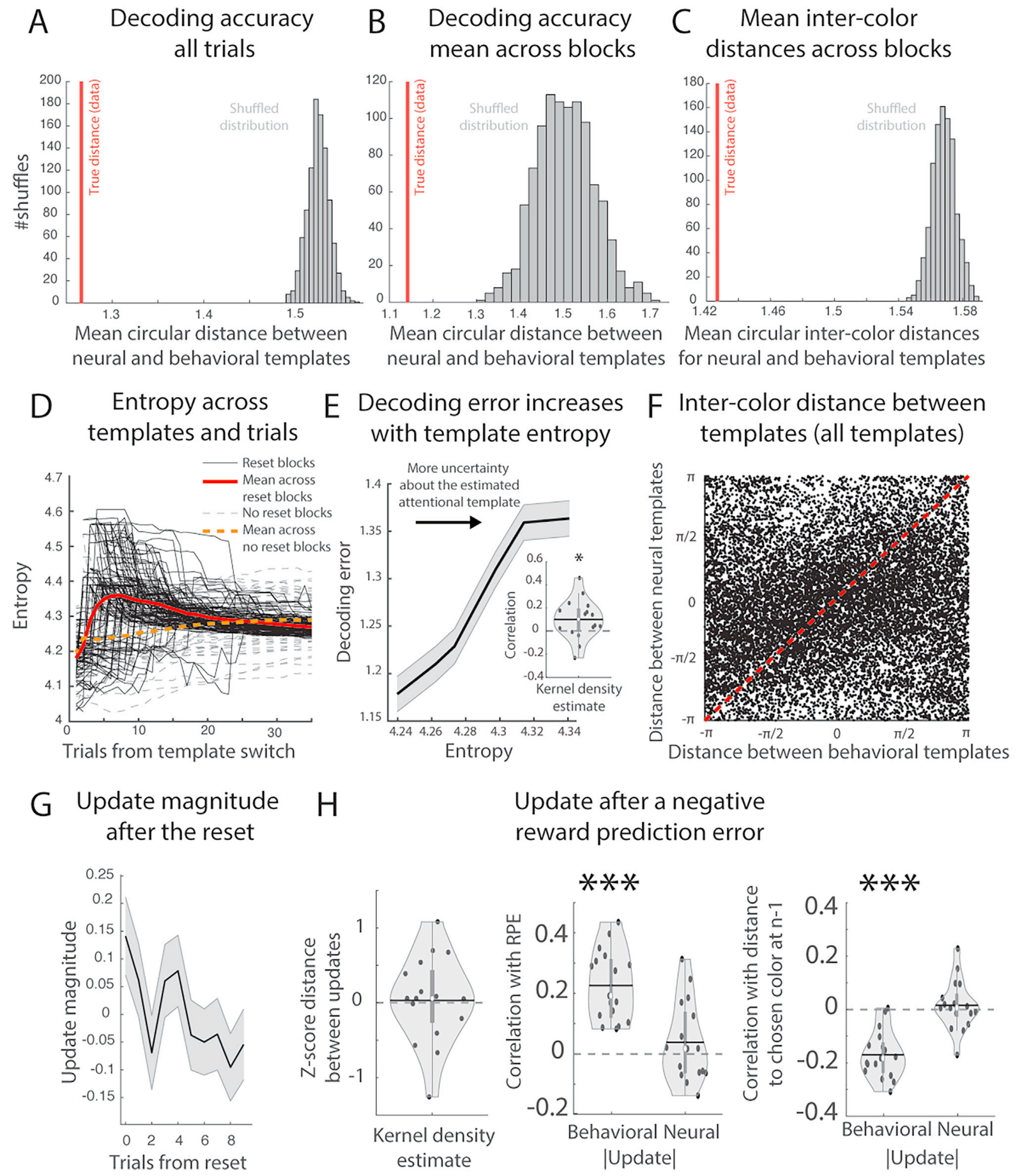

Figure S4. Extended analysis of the structured and incremental nature of the estimated template representation, related to Figures 3 and 4

(A) Results of the permutation test on the trial-by-trial decoding accuracy for all trials. Gray bars represent the histogram of the shuffled mean circular distance between the neural and behavioral templates (1,000 shuffles, the smaller the distance, the better the decoding accuracy), and red line represent the true mean circular distance between the neural and behavioral templates across all trials.

(B) Same as (A), but for the decoding accuracy across blocks (related to Figure 3D).

(legend continued on next page)

(C) Same as (A), but for the mean circular distance of the inter-color distance between two neural templates and two behavioral templates (related to Figure 3E).
(D) Entropy of attentional template over trials, relative to template switch. Each line is a series of trials after the template switch (171 blocks). Entropy is often low before monkeys realize the template has changed.
(E) Decoding error measured as absolute circular distance between neural and behavioral estimated templates (± SEM) for each bin of entropy (see STAR Methods, 10 bins, smoothing of 4 bins, all withheld trials). Violin plot shows mean Pearson correlation between the decoding error and the template entropy across validation trials for each session. The greater the attentional entropy, and therefore the uncertainty about the estimated template, the greater the decoding error ($r(5,838) = 0.0954$, $p < 0.001$, one-sided Pearson correlation). This effect was consistent across sessions ($t(16) = 2.4996$, $p = 0.0118$, one-sided t test).
(F) Scatter plot of circular distance between mean neural and behavioral estimated templates ($r = 0.1092$, $p < 0.001$, 14,535 pairs, permutation test on the circular distance). G) Mean absolute angular distance between neural estimated template at trial n and n+1 over trials, relative to the reset (mean across blocks, ± SEM, 111 blocks). The update magnitude decreased after the reset (GLM with factor trial after the reset, $\beta = -0.019 \pm 0.007$, $t(1,108) = -2.6863$, $p = 0.007$). H) Left: mean *Z* scored distance between the neural and the behavioral update away from the chosen color on withheld trials, following a negative RPE. Black dots are individual sessions, central white dot is the median, horizontal black bar is the mean, thick vertical gray bar represents the 25th to 75th quartile, and area represents the kernel density estimate of the data. Middle: mean Pearson correlation between the update magnitude and the RPE magnitude on withheld trials. Right: same as middle but for absolute distance between the estimated template before the update (at n-1) and the chosen color. For all panels, ***$p \leq 0.001$.

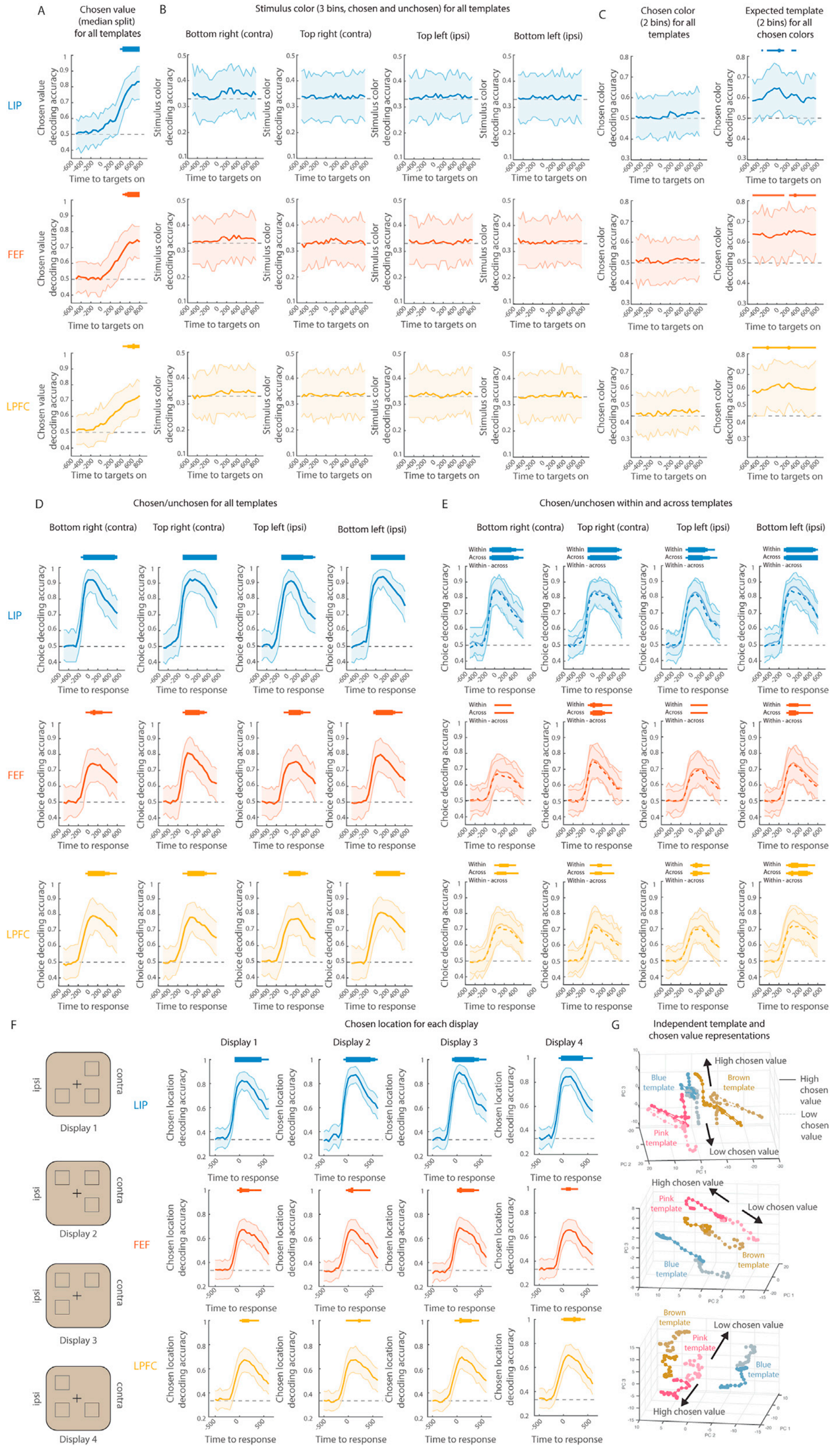

(legend on next page)

CellPress
OPEN ACCESS

Figure S5. Extended analysis of stimuli re-mapping to support decision-making across templates, related to Figure 5

(A) Time course of classification accuracy of chosen value (with 95% confidence interval; 110 neurons, trained/tested on 80%/20% of 128 trials per estimated template bin, 3 bins). Chance level was 1/2. For all panels, bar thickness indicates significance level: $p \leq 0.01$, $p \leq 0.05$ Bonferroni corrected (27 time points), and $p \leq 0.01$ Bonferroni corrected.

(B) Time course of stimulus color classification accuracy of stimulus color (with 95% confidence interval). Computed on withheld trials for each time point (115 neurons, trained/tested on 80%/20% of 128 trials per stimulus color bin, 3 bins; split between chosen and unchosen stimuli). Chance level was 1/3. $P \geq 0.23$ for all three regions.

(C) Time course of classification accuracy of chosen color (left) and estimated template color (right; both with 95% confidence interval; 133 neurons, 80 trials per chosen color and estimated template bin, 2 bins for each). The chosen color could not be decoded when we balanced the estimated template color (all $p \geq 0.28$), whereas the estimated template could be decoded when we balanced the chosen color ($p \leq 0.009$ in all three regions, although this did not survive Bonferroni correction. For this panel, bar thickness indicates significance level: $p \leq 0.05$, $p \leq 0.01$, and $p \leq 0.05$ Bonferroni corrected across time (27 time points).

(D) Time course of classification accuracy of choice (with 95% confidence interval; on withheld trials; 90 neurons, trained/tested on 80%/20% of 120 trials per estimated template bin, 3 bins). Chance level was1/2. Bar thickness indicates significance level: $p \leq 0.01$, $p \leq 0.05$ Bonferroni corrected (23 time points and 4 locations), and $p \leq 0.01$ Bonferroni corrected.

(E) Same as (D) but computed on withheld trials with the same estimated template color as the training trials (solid line) or with a different estimated template color bin (dashed line).

(F) Time course of classification accuracy of choice location in the four possible displays (3 targets were presented in the 4 possible locations on each trial) (with 95% confidence interval; on withheld trials; 105 neurons, trained/tested on 62 trials per estimated template bin, 3 bins). Chance level was 1/3. Bar thickness indicates significance level: $p \leq 0.01$, $p \leq 0.05$ Bonferroni corrected (23 time points and 4 displays), and $p \leq 0.01$ Bonferroni corrected.

(G) Neural activity in all three regions projected into a reduced dimensionality space consisting of the first three eigenvectors (in decreasing order of explained variance). LIP, 145 neurons; FEF, 212 neurons; LPFC, 474 neurons.

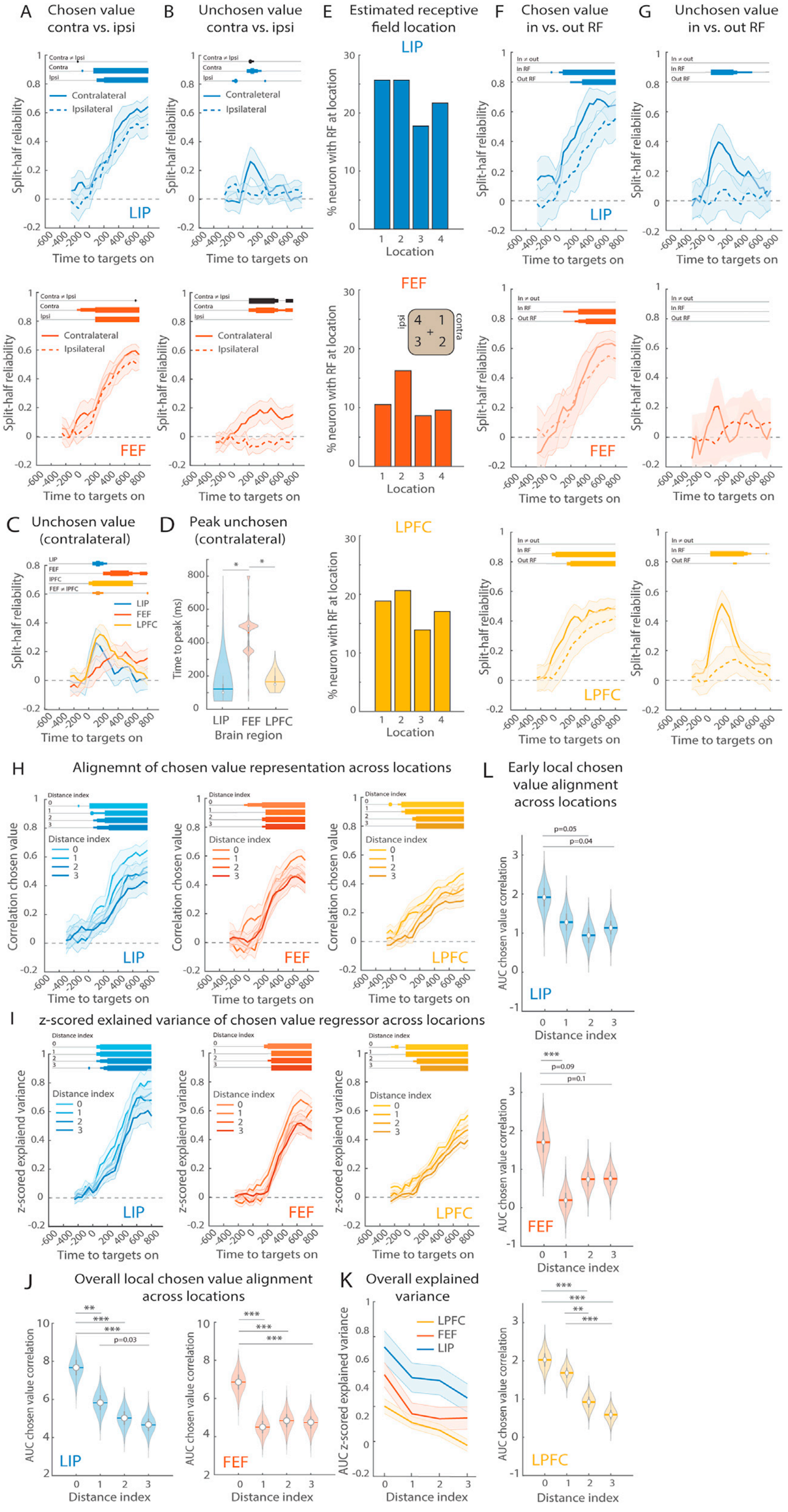

(legend on next page)

CellPress
OPEN ACCESS

Figure S6. Extended analysis of value representations over time, related to Figure 6

(A) Time course of the mean split-half reliability (with 95% confidence interval) of the chosen value regressors for the two contralateral locations (contra) and the two ipsilateral locations (ipsi, dashed line) for LIP (top) and FEF (bottom) (see Figure 6A for LPFC). Bars indicate the significance of the split-half reliability (blue/red) and the difference in reliability strengths between the two regressors (black).

(B) Same as (A) for the unchosen value for LIP and FEF (see Figure 6B for LPFC).

(C) Same as (A) for the unchosen value regressor in the contralateral locations for LIP, FEF, and LPFC. Bars indicate the significance of the split-half reliability and the difference in reliability strengths between FEF and LPFC (orange). There was no difference between FEF and LIP ($p \geq 0.0324$) and LIP and LPFC ($p \geq 0.0102$).

(D) Violin plot: time point at which the unchosen value reliability was maximal across bootstraps. Central white dot is the median, horizontal bar is the mean, thick vertical gray bar represents the 25th to 75th quartile, and area represents the kernel density estimate of the data. Statistical significance was estimated using a two-sample two-tail z test. $^*p \leq 0.05$.

(E) Percent neurons with a firing rate modulated by the presence or absence of a stimulus at each location, “in” receptive field, note that a neuron can have several locations in its receptive field.

(F) Same as (A), but comparing locations “in” receptive field and “out” of the receptive field (defined in E). We subsampled the locations “out” of receptive field to match the “in” receptive field.

(G) Same as (B) for “in” and “out” of receptive field.

(H) Time course of the correlation of chosen value regressors within a location (i.e., split-half reliability, distance index = 0) or across locations (distance indexes > 0; see STAR Methods for details). Shown for LIP (left), FEF (middle), and LPFC (right). Shaded region shows 95% confidence interval. Bars indicate the significance of the split-half reliability (distance index = 0) and the correlation disattenuation between chosen value vectors.

(I) Same as (H), but for the *Z* scored explained variance of the chosen value regressor (see STAR Methods for details).

(J) Bootstrapped distribution of the mean area under the curve of the correlation of chosen value regressors within a location (split-half reliability, distance index = 0) or across locations (distance index > 0). Taken for all (22) time points shown in (H). Shown for LIP (left) and FEF (right). Central white dot is the median, horizontal bar is the mean, thick vertical gray bar represents the 25th to 75th quartile, and area represents the kernel density estimate of the data. Statistical significance was estimated using a paired one-sided z test (assuming that smaller distances would be more similar) using the reliability or the correlation disattenuation against 0. $^*p \leq 0.05$, $^{**}p \leq 0.01$, $^{***}p \leq 0.001$ Bonferroni corrected (6 pairs).

(K) Mean area under the curve of the *Z* scored explained variance of the chosen value regressor (shown in I, see STAR Methods for details).

(L) Same as (J) but limited to time points up to 300 ms after the onset of the stimuli in (H) (12 time points).
